# Computer algebra in gravity research

**DOI:** 10.1007/s41114-018-0015-6

**Published:** 2018-08-20

**Authors:** Malcolm A. H. MacCallum

**Affiliations:** School of Mathematical Sciences, Queen Mary University of London, Mile End Road, London, E1 4NS UK

**Keywords:** Computer algebra, General relativity, Gravitation theory, Algorithms, Exact solutions, Programs

## Abstract

The complicated nature of calculations in general relativity was one of the driving forces in the early development of computer algebra (CA). CA has become widely used in gravity research (GR) and its use can be expected to grow further. Here the general nature of computer algebra is discussed, along with some aspects of CA system design; features particular to GR’s requirements are considered; information on packages for CA in GR is provided, both for those packages currently available and for their predecessors; and applications of CA in GR are outlined.

## Introduction

The term “computer algebra” (CA) covers the theory and implementation of computer programs to perform the symbolic manipulations and calculations usual in mathematics, in particular those arising in algebra and calculus. Fuller descriptions are given in texts cited below. This review deals with the use of computer algebra in gravity research (GR) in the period 1980 onwards: work before that date is described in cited earlier reviews. Because of the complicated nature of calculations in GR, CA methods have proved very useful in many aspects of GR research.

In the following sections, I discuss the nature of CA, the requirements of its use in GR, and the available programs. Finally a (hopefully representative) selection of specific applications, from the many made, is described. A fuller description of the contents of the main sections below is given in the next subsection and Sect. 1.5.

### Computer algebra

This would be a very long review if it attempted to cover all aspects of the design, implementation and features of computer algebra (CA) systems and their history, and then similar details for all packages for application in gravity research (GR: this abbreviation could be understood more narrowly as “general relativity”, but here the wider meaning is intended). Moreover there are now good textbooks which describe algorithms and general design features of CA systems,^1^ e.g. Geddes et al. (1992), Mignotte (1992), Davenport et al. (1993), Bronstein (1997), Cohen (2002), von zur Gathen and Gerhard (2013) and Bostan et al. (2017); there were few such texts before 1990. There are also texts discussing the use of CA in physics (e.g. Grozin 1997; Enns and McGuire 2001; Baumann 2005) and one that contained introductions to three of the main CA in GR systems of the day (MacCallum et al. 1994). There have been numerous journal reviews of CA in GR, many cited below.

CA itself is part of the wider field of symbolic computation. Current developments are discussed in the specialist journals “ACM Communications in Computer Algebra” (and its predecessor the “ACM SIGSAM Bulletin”), published by the Association of Computing Machinery (ACM) Special Interest Group on Symbolic and Algebraic Computation (SIGSAM), “Applicable Algebra in Engineering, Communication and Computing”, and “Journal of Symbolic Computation”. The ACM journal also provides news of activities in the CA research community. There are specialist conferences, listed at [C69], notably ACM SIGSAM’s annual ISSAC (International Symposium on Symbolic and Algebraic Computation), whose proceedings contain many important papers; papers on CA are also spread widely throughout the mathematical and computer science literature.

As well as journals and conferences, there are a considerable number of books and papers devoted to specific systems or applications or both: I shall mention some of those which describe systems widely used in GR, or describe particular GR uses, later, and give pointers to the more extensive online bibliographies available. Where it may be of interest or help to the reader but is not mentioned directly in the text I have appended to a citation the name of the CA package used, in, e.g., the following form (Stephani et al. 2003 {Classi}). Appendix A gives a alphabetical list of the packages showing where in this review further information can be found.

Because many packages and the information about them are now made available only via the Internet rather than published in books or on CDs or DVDs, I have provided Appendix C in which the URLs for downloads of, or information on, systems and packages can be found: references to the data there have the form, e.g., [C61].

Three starting points are often cited as beginning the history of CA. The earliest (not counting the prescient 19th century arguments by Ada Lovelace and Charles Babbage that such computations should be possible with what we now call computers), and most frequently cited as the starting point, was work by Kahrimanian (1953) and Nolan (1953) in which symbolic differentiation was implemented. Next, and sometimes cited as the first full CA use, was the paper by Boys et al. (1956). They used EDSAC (the early computer built at Cambridge University) saying “the advent of such machines has simplified and made practical such calculations, not only as a means for performing the arithmetical operations, but also for the carrying out of much of the mathematical analysis of the most formal type”. Their application involved antisymmetric functions “formed from a set of elementary three-dimensional exponential polynomial functions”, leading in some cases to “formulae involving more than five hundred terms”. A further starting point sometimes cited is McCarthy’s invention of Lisp, which proved a suitable implementation language, as described below. Development of CA was slow for quite a while, awaiting improvements in hardware, implementation languages, and systems software.

The first more complete system was FORMAC (Bond et al. 1964), whose development began in 1962; it was based on the programming language widely used in the 1960s for numerical computation, FORTRAN. Because FORTRAN was not very suitable for algebraic manipulation, FORMAC was later rewritten in PL/1 (Xenakis 1971). A second major system was REDUCE, stimulated by McCarthy’s proposal of Lisp as a basis for non-numerical calculations in physics (Hearn 2005) [C67], and initially intended and used for computations in quantum electrodynamics (Hearn 1966). A third, developed from 1968 to 1969 onward as part of Project MAC at the Massachusetts Institute of Technology was Macsyma (MACSYMA is short for Project MAC SYmbolic MAnipulator). [This followed an earlier Lisp-based system at MIT called MATHLAB (Ogilvie 1989).] Macsyma and Reduce were (and are) based on (different dialects of) Lisp.

Many other systems were developed at that time (see Moses 2012) and since. van Hulzen and Calmet (1983) reported an estimate that there were then about 60 systems, and the 2003 handbook (Grabmeier et al. 2003) describes 9 general purpose systems, 43 special purpose systems and 15 packages. As of February 2018 the Wikipedia^2^ page comparing CA programs [C80] listed 39. The swMATH project has analysed more than 215,000 citations of software and from them lists the citations of more than 100 CA systems (Heinle et al. 2017) [C62]. Of these many systems, some were or are specialized, for example, to commutative algebra or polynomial rings, and so were or are not generally applicable in gravity research. This being a review of CA in gravity research, only those systems that have been or could be used for such purposes will be mentioned.

Yun and Stoutemyer (1980), Gerdt et al. (1980) and van Hulzen and Calmet (1983) give useful surveys of the early systems. Among those systems, Macsyma and Reduce have been the most used in GR. The most important post-1980 systems for such applications are Maple and Mathematica$^{\textregistered }$. Maple and Mathematica$^{\textregistered }$ are also today’s most successful and widespread commercially available systems (whereas both Macsyma, in its Maxima incarnation, and Reduce are now open source). These systems are discussed in more detail in Sect. 3, though some aspects of their designs will be referred to in the preceding Sect. 2, which deals with general considerations in the design and implementation of CA systems.

As well as these four main general purpose systems for which packages for relativity have been written (Macsyma, Maple, Mathematica$^{\textregistered }$ and Reduce), I shall describe in Sect. 3 below some others which either had interesting architecture or were employed in interesting GR applications.

In Sects. 6 and 7 I list and briefly describe systems or packages written since 1980 which I know to have been used or intended for use in GR or related fields. (I also give references to further earlier surveys of CA in GR.) Some of those systems and packages have ceased to be available or be maintained or developed, or have become obsolete or otherwise defunct: they may therefore not be currently used or even usable. Despite that, information about them may make papers which used them in applications more intelligible. I shall refer to them just as “earlier”, since I am not sure in all cases of the current status. Some may still be available and usable, and in a few cases that is definitely so, even though the packages may have had no recent development or maintenance. I have tried to indicate where this is the case (it applies, for instance, to the Maple package GRTensorII and the Reduce package GRLIB).

In addition, there are also a number of packages (Hartmann and Davis 1989; Ilyin and Kryukov 1989; Seiler 1991; Jamin and Lautenbacher 1993; Poghosyan 2005; Abłamowicz and Fauser 2015) which provide facilities for Clifford algebras or more specifically for $\gamma$-matrix algebra. Such packages can be used both in quantum field theoretic and geometric calculations, but were considered too specialized to be included in Sects. 6 and 7: many of their applications fall outside GR.

### Pros and cons of CA

The main reasons for using computer algebra (CA) are speed and accuracy, the ability to handle more complicated calculations than can be done by hand, and relief from the boredom of repetitive tasks. These all apply to applications in GR, so much so that GR, celestial mechanics and quantum electrodynamics supplied much of the early motivation for developers (Yun and Stoutemyer 1980; van Hulzen and Calmet 1983); Barton and Fitch (1972) gave a survey covering early use in all those three areas.

In more detail, the advantages of computer algebra are as follows.

1. Accuracy. CA gives exact and accurate answers: well-written and tested computer algebra packages do not lose factors of 2 or get the sign wrong.
2. Speed. CA is fast: the classic example is Delaunay’s calculations of lunar motion in the 19th century, which took him 20 years, but was repeated and corrected (using at least 18 h of IBM 360-44 CPU time) by Deprit et al. (1970). An example in relativity is given by the famous Bondi metric, where the initial calculations (Bondi et al. 1962) took 6 months and still contained errors, whereas d’Inverno’s clam could repeat the calculation (correctly!) in 18 s (see Table 3 of d’Inverno 1980), and my PC now does it in milliseconds.
3. Repetitive tasks. CA removes the tediousness of almost identical repeated calculations, for example calculations of curvature for similar metrics.
4. Calculations infeasible by hand. The power of the systems opens up projects which would be unthinkable by hand, sometimes with unexpected results. For example, a brute force calculation may lead to a simple answer which prompts new insight, revealing deeper principles and enabling the answer to be derived more elegantly (e.g. Frick 1977a; Pavelle 1979; Amerighi et al. 1986 {Sheep, Macsyma, Stensor}). A more recent example is given by Torre (2012) {DifferentialGeometry}, where a result is given that was only obtained while testing some “fairly elaborate code” (in the author’s words).

The last two points are especially relevant to applications in gravity theory. As already mentioned, GR was a testing ground of many early computer algebra systems, essentially because of the large size of the Einstein equations for general relativity expressed in terms of a general metric in coordinate form, which stretched those systems to their limits. There are 4 equations with around^3^ 8574 terms and 6 with 11,018 terms: these numbers themselves come from a CA calculation.

I should add a warning note here about the last advantage. Despite the enormous increases in CPU speed and storage capacity since CA began, the limitations on practical uses of CA still come from computation time and storage requirements. CA can successfully tackle expressions with hundreds of thousands or even millions of terms (though the value of the output if the expressions do not simplify to a small number of terms seems rather doubtful), but combinatorial explosions quickly outrun the capacity of any system. It remains quite easy to set up calculations which completely fill available memory. (One symptom can be excessive paging to and from virtual memory or swap space. This occurs when the main memory becomes full and the operating system swaps some data out and replaces it, only to find it needs, and reads back, the copied data, which then fills the main memory again ...Some systems, such as muTENSOR, FORM and Stensor, had a system of “bucketing”, or writing to disk, designed to limit that problem.)

For instance, the number of terms in the determinant of an $n \times n$ matrix each of whose entries is a sum of 2 terms is $2^nn!$ (before any collection of like terms). This grows rapidly with *n*, and most general purpose algebra systems on an average machine become unable to cope at about $n=10$–11, which often surprises unwary users who think that since they can handle $n=3$ easily and are not daunted by $n=4$, a machine should be able to do much more. A reasonable estimate, due to Fritz Schwarz, of the increase in the size of expressions which can be handled by using CA instead of “hand calculation” is a factor of $10^4$, or maybe $10^5$.

Advanced algorithms can prove hard to implement in an efficient manner, and some CA algorithms have long running times, so time, as well as the memory requirements of large expressions, can still be a limiting factor. Good complexity estimates would help in estimating requirements, but in practice one often just has to try things out. Both for timing comparisons and absolute values of timings, the information in past papers is likely to be long outdated due to changes in hardware, systems software and CA systems. Comparative data are still of interest but an up-to-date experiment to check them might be wise.

The reasons *against* using CA include the cost, in time and effort, of learning one or more systems and the possibility of encountering bugs. But perhaps the worst hurdle for many users is the need to develop a sufficient understanding of the underlying mathematics to be able to anticipate where the system might give no answer or a wrong answer: it has been recognized for a long time that not only designers and implementers need this understanding (van Hulzen and Calmet 1983). The fault may not be with the implementations available, but with the lack of any, or any efficient, general algorithm. One could argue that CA systems ought not to attempt to cover calculations which lack a good algorithm. In practice, user or commercial pressures may dictate that when there is no algorithm, heuristics, that by definition may not always work, are used instead.

Among the areas where good general algorithms are hard to find, or non-existent, are problems in handling the following: algebraic numbers; functions with branch cuts (Dingle and Fateman 1994); definite integrals requiring contour integration; and simplification using identities involving sums (e.g. given a polynomial in $\sin x$ and $\cos x$, return the shortest equivalent expression obtainable by using the identity $\sin ^2 x+\cos ^2 x=1$, i.e. the one with fewest completely expanded terms to be added together).

This last is the problem of “sum-substitution”. Although there are formal methods, such as Gröbner bases (Buchberger and Winkler 1998), to find a canonical representative in a space of equivalent polynomial expressions, in a sense specified by some well-defined term ordering, that representative may be far from being the one the user would wish to obtain, e.g. the shortest possible equivalent expression. This applies not only in the trigonometry example above, but (e.g.) in the context of the indicial tensor calculators discussed in Sect. 4. Automating the choice of where, and where not, to make a sum-substitution, gives difficulties (Hörnfeldt 1979): one may not wish to apply the same substitution for, say, $A+B$, to all expressions containing $A+B$.

The procedure used by Stensor (Hörnfeldt 1977, 1979) seems by experiment to be rather effective but is not, as far as I know, fully documented anywhere: to obtain a full description one would need to analyse the rather complex code itself (which was said in 1989 to have required 14 years of work resulting in code 2/3rd of the length of the Reduce source code). There is a presentation in Hörnfeldt (1979) and a description of its use as a simplifier for trigonometric expressions (Hörnfeldt 1982), stating that it considers expressions of the form $a \sin ^n x \cos ^m x$, where *a* is a numerical coefficient, by considering reduction not only within the triangle of terms with powers (*m*, *n*), $(m-2, n+2)$ and $(m-2, n)$ but also the terms with powers $(m-4, n+4)$, $(m-4, n+2)$ and $(m-4,n)$. Presumably similar ideas were used in more general cases.

There is a high cost in time and effort in writing one’s own system or package, whether general or specifically for GR (although, as this review shows, the latter has been a popular endeavour). Even 30 years ago, it was said that to write a passable general-purpose system required at least 50 person-years of effort. To match existing systems now would impose a much higher requirement, despite the help provided by the books and articles now available which describe efficient implementations of CA algorithms, because the systems have been extended substantially both by their user communities and, for the proprietary systems, by the companies that own them. For all the larger systems, the total number of contributors is large.

When writing a package specifically for use in GR there is a considerable added risk of “reinventing the wheel”, given the number of packages there have been (failure to re-invent the wheel can of course be costly in a different way). There are about 100 packages listed in the table in Appendix A, the exact number depending on what is counted as a distinct package, and the list is almost certainly incomplete. (This is more than double the number of such packages I knew of before starting to compile this review.) It is rare for authors of packages to explain in what way their package offers new internal design features of value, which makes understanding the real differences more difficult: instead the focus of authors’ descriptions tends to be on the set of calculations a user is enabled to perform. I would strongly advise anyone considering writing a new package to first delve into the details of existing packages. For example, if one wishes to perform a particular calculation but does not know of an existing package that provides that facility, it may be much simpler to add that facility to an appropriately chosen package than to build a new package.

### CA in GR

Gravity research covers a wide area and CA may be useful not only to theorists but also to researchers whose primary interests are experimental or numerical. In principle it of course includes Newtonian gravitational calculations, e.g. in astronomy, and Newtonian and relativistic celestial mechanics, but I have not attempted to survey those applications. Predicting the paths of spacecraft is an important engineering application of general relativity. Thus relativistic celestial mechanics programs can have practical as well as astronomical uses. Accurate numerical values for orbits, however, generally come from numerical rather than algebraic programs. For some developments of CA in celestial mechanics later than those mentioned above see Brumberg et al. (1989), Deprit and Deprit (1990), Vakhidov (2000) and Laskar and Gastineau (2012). However, I am told (Carl Murray, private communication) that celestial mechanics researchers now tend to use packages in general purpose CA systems rather than special purpose programs.

Because of the close connection between GR and differential geometry, I shall include mention of CA systems and packages for differential geometry, as these can easily be specialized to, for example, spacetimes in general relativity. However, I shall not cover packages designed mainly for three-dimensional vector calculus in curvilinear coordinates or for the geometry of surfaces in three-dimensional space, for studying or solving systems of differential equations (some interfaces to which are mentioned by Dray 1996) nor, in general, those for evaluating Feynman diagrams or for similar applications in quantum field theory, although such systems can be useful for work on quantum field theory in curved space or quantum gravity (which I shall refer to collectively as quantum GR).

Moreover I note that searches for tensor software on the internet will find, in addition to the sorts of package described here, packages or libraries aimed at numerical simulations in, for example, non-relativistic fluid dynamics or elasticity, which are disciplines making heavy use of tensors in flat space, often in curvilinear coordinates. (Conversely, CA systems which handle curved spaces can be used for calculations in such disciplines: see for example Bebbington and Göbel 2001 and Sect. 7.4.)

Under the hardware constraints of the 1960s, specialized systems for GR were almost a necessity, though FORMAC was used quite a bit in early work [beginning with work of Thorne and Zimmerman, of Clements and Matzner, and of Ernst (d’Inverno 1980)]. Other early systems were GRAD Assistant (Fletcher et al. 1967), based on Lisp, CAMAL, the CAMbridge ALgebra system^4^ (Wainwright 1978; Fitch and Cohen 1979; Fitch 2009), Lisp Algebraic Manipulator (LAM) which was originally implemented at Kings College London in Lisp and in machine code (d’Inverno 1969; d’Inverno and Russell-Clark 1974), and packages or programs in Macsyma and Reduce. In total, quite a number of packages were written in those early years: for example, d’Inverno (1980) mentions 7 in FORMAC alone. The early packages, and their applications, were surveyed by Barton and Fitch (1971), d’Inverno (1975, 1980, 1983), Cohen et al. (1976, 1984), Pavelle (1979) and Ogilvie (1989), and earlier reviews cited therein. Work behind the “Iron Curtain” is discussed in Gerdt et al. (1980) and Fedorova et al. (1989). As many early packages were of limited capability compared with present-day ones, due both to improvements in computer power and improvements in software design, I shall not repeat the information from those reviews, instead concentrating on work since 1980.

Those early systems were written mainly in FORTRAN or PL/1 (for FORMAC), or Lisp (for Macsyma and Reduce), or in assembler or native languages for particular machines which are now only available in museums. Examples of machine-specific systems were ALAM for Atlas (d’Inverno 1969), CLAM for CDC 6600 (d’Inverno and Russell-Clark 1974), the TITAN and IBM machine language versions of CAMAL (CAMAL was later written in BCPL, the forerunner of C, which was first introduced in 1978, see Kernighan and Ritchie 1988) and the first versions of Sheep (now called Sheep1), written in the macro language for DEC-10 machines. Indeed d’Inverno (1980) pointed out that at that time all the systems only ran on specific hardware.

It should be noted that the implementation languages for CA systems and packages (such as FORMAC, Lisp, C, and Python^5^) are rarely also the programming languages presented to the user by those systems.

There are several reasons for CA packages for GR aging or dying. Most such packages are developed and maintained by only small groups of people, and lack a sufficient user base to sustain themselves financially (or justify owners of general purpose systems putting resource into maintenance of GR packages).^6^

Even those packages that have been maintained may accrete code from several different stages of evolution of the underlying systems and thus end up with parts in different styles or requiring different facilities, potentially giving rise to problems. (I have been told this applies to, for example, xAct, described in Sect. 6.3.4.)

Some packages have died because of changes to the underlying general purpose algebra system (“general purpose” as described in Sect. 3), which discouraged the package’s originator from updating. Significant changes, such as when Reduce (or more accurately its underlying Lisp) switched from upper case as default to lower case, are sometimes called “flag days”, and can make packages unusable unless their maintainers are willing to invest significant effort. As examples of this effect, RicciR (Kadlecsik 1996) ceased development due to changes between Reduce 3.5 and later versions, and GRtensorII [C24] was not updated for some time after Maple version 11 (it can still be run, and was recently replaced by GRTensorIII).

Changes are not always negative, however. Additional facilities in, or other improvements to, a general purpose system may stimulate or facilitate progress in CA software. For example, Maple’s good algorithms for handling over-determined systems of linear partial differential equations, such as often appear in differential geometric applications, are used by DifferentialGeometry (see Sect. 6.2) in finding symmetry generators, geometric objects with a prescribed group of symmetries, parallel tensor fields, and so forth, and were also used by Hickman and Yazdan (2017).

Mathematica$^{\textregistered }$ and Maple now have annual licencing, and Maple in particular has recently had annual updates, with the consequence that it takes more and more effort for package maintainers (and users!) to keep up, as well as making it difficult or impossible, when annual licences expire, for one to test packages which worked under previous versions, even if one has a licence for the current version. (Reduce is even more frequently revised, see Sect. 3.1.4, but generally not in such a manner as to break existing packages.) I have not attempted to check whether currently available packages all work with the current system versions, but in Sect. 6 I have noted available information about the latest versions for which I have found that packages are, or claim to have been, updated.

A second reason packages die is change of personnel. In extreme cases this may be due to the literal death of the lead author, but more commonly it is because people move from academia to other employers, or shift research topics, perhaps because their CA in GR work does not attract sufficient recognition. (Software design and implementation tends to be undervalued in academia by those who do not do it, despite its need for original and ingenious ideas.^7^)

Lastly, because some CA systems are now produced by substantial companies, packages or systems may cease to be maintained due to commercial pressures.

### Choosing a CA package

Before any discussion of CA’s nature, designs, and capabilities, or its applications in GR, I want to state firmly that *there is no best package*, either for CA in general or CA in GR in particular. If you have found a package which works with the machine and operating system, or within the general purpose CA system you are already familiar with, and which does the calculations you want to do, or can readily be adapted to do so, use that. And if you have not found such a package (yet), look for one such rather than seek a “best” one. In the final section of this review I say a little more on this issue, despite the cautionary remarks that follow.

In particular, do not believe anyone, including me, who purports to draw objective comparisons, without making your own independent check. Users accustomed to particular systems often offer comparisons based on incomplete knowledge either of the other available systems or of the underlying mathematics and computer science. They tend to focus on the good points of the systems they know well, and criticise other systems which do the same things less well or not at all, while disregarding the strong points of those other systems. (Claims that the author’s favoured system is the only one that can do a certain type of calculation should be viewed with particular scepticism, although they may sometimes be correct.) One can hence find examples where system A was better than system B in one comparison and B surpassed A in another. Moreover, all such comparisons go out of date very fast.

That warning should be followed by a “full disclosure” or “disclaimer” to help the reader to discount any bias that may be present in the following review. The systems with which I am most familiar are Reduce (see Sect. 3.1.4) and Sheep (see Sect. 7.4), for both of which I have written introductions (MacCallum and Wright 1991; MacCallum et al. 1994) and contributed code, and Maple, on which I have taught courses. At the time of writing, my own computer can run under either Windows and Linux: in addition to Reduce and Sheep it runs recent versions of Axiom, Cadabra, Maxima,^8^ Maple, Mathematica$^{\textregistered }$ and Sage, and I have downloaded and briefly tested a number but not all of the CA in GR packages listed as currently available for those systems in Sects. 6 and 7.

One should also note that while several, or even many, systems may be able to deal with a particular problem, they will vary in their hardware requirements or efficiency. As an essentially historic aspect, due to the increases in speed and memory of machines, some systems had important built-in limits on the numbers or sizes of objects. As examples, Reduce 3.5 and Maple V.3 on DOS-based PCs had limitations of this sort which prevented certain calculations, feasible on other hardware, from being carried out.

### Plan and aims of this review

In Sect. 2 I discuss design issues in CA in general, without much specific relevance to GR. This is followed by a survey of available general purpose systems for CA in Sect. 3. I consider what calculations are required in GR in Sect. 4. The resulting design considerations for CA in GR are introduced in Sect. 5, and this is followed by two sections listing and briefly describing CA packages for use in GR, firstly those built on general purpose CA systems, in Sect. 6, and then those which are stand-alone, in Sect. 7. I conclude with a section discussing applications, Sect. 8, and appendices giving an alphabetic cross-listing of packages, a note on packages with some selected special features, and the list of URLs quoted in the text.

I have tried to give rather complete lists in Sects. 6 and 7, omitting only the many early packages discussed in the reviews cited above which I believe to be no longer maintained or no longer available, and some others for which I could not find any current online or printed documentation. Other such lists have been provided by authors of some of the packages, for example at [C23] and [C38]: see also [C80] and [C62].

In each of Sects. 6 and 7, I have included “earlier” subsections for the earlier packages as defined above. One particular source of packages is the journal ‘Computer Physics Communications’, which maintains an archive [C71] of the programs presented in articles. Although some of those in the archive were frozen at the time of their publication, others are still maintained and/or distributed. I have included below all those packages which I believe to be still of use now or to have been used in the past in interesting applications.

The reader will infer from the large number of packages mentioned and their dispersion in the literature that it is highly likely that there are further packages which I am not aware of. Indeed some of those described are only available via the internet, without related published books or articles: as already stated I have tried to provide the relevant URLs.

I have not attempted to list for each package the facilities it has or lacks. That is described in the individual packages’ manuals, which together cover thousands of pages. Trying to make any useful digest would thus take an enormous amount of effort, still fill many pages, and be inevitably unreliable. Any attempt to do it is made more difficult by the advent of “worksheet” or “notebook” interfaces, online tutorials, and online manuals accessed from within the system, replacing the more common printed or printable manuals ubiquitous in the past; those newer resources mean one has to run the system in order to see what is available.

I therefore instead give only a general indication of the nature of each package together with references enabling readers to find out more. If presented as quotations, the short descriptions come from the packages’ own material. Some other descriptions are based on freely available digests made by others. I apologize to anyone whose work has unintentionally not been adequately credited.

Despite those cautionary remarks, I have added Appendix B, providing a list (probably very incomplete) of packages offering certain important specific types of calculation, for example listing packages providing the Newman–Penrose formalism.

The section discussing applications, Sect. 8, is as complete as I could make it, in terms of the topics in GR mentioned, but certainly does not provide a comprehensive bibliography of papers in GR where CA was used, if only because researchers may use a CA package in their calculations without mentioning it in the published work (d’Inverno 1980 gives examples from his own research). However, I shall be very pleased to hear about applications which are not covered by one of the subsections in Sect. 8, and papers which make substantial advances in the areas I do mention. I hope the selected examples will assist readers considering work on a particular application and looking for some related work as a starting point.

Although this review covers gravity research rather than just general relativity, many applications are within that theory or closely-related generalizations. In discussing such work I shall assume except where otherwise stated the same notational and sign conventions as in Stephani et al. (2003), the most important of which are that the 4-dimensional spacetime signature is taken to be $(+\, +\, +\, -)$; lower case Latin indices run from 1 to 4, with any timelike coordinate last; a comma denotes partial differentiation and a semicolon covariant differentiation; symmetrization and antisymmetrization of index pairs are indicated by round and square brackets respectively: thus

$$\begin{aligned} v_{(ab)} := {\frac{1}{2}}(v_{ab} + v_{ba}), \quad v_{[ab]} := {\frac{1}{2}}(v_{ab} - v_{ba}). \end{aligned}$$

The Riemann tensor is defined by $2v_{a;[bc]} = v_d {R^d}_{abc}$, for any vector field **v**, and obeys the first Bianchi identity

1 $$\begin{aligned} R_{a[bcd]} = 0 \Leftrightarrow R_{abcd} + R_{acdb}+R_{adbc} =0, \end{aligned}$$

and the Ricci tensor, Einstein tensor, and scalar curvature are defined by

$$\begin{aligned} R_{ab} := {R^c}_{acb}, ~~ G_{ab} := R_{ab} - {\frac{1}{2}}Rg_{ab},~~ R := {R^a}_a. \end{aligned}$$

Other conventions are defined later as required.

For clarity later a number of (hand) computation techniques in relativity need to be defined. To avoid writing a whole new textbook explaining these I shall usually refer to the relevant chapter or section of Part I of Stephani et al. (2003), but good accounts of each of them can be found in many other places (e.g. Penrose and Rindler 1984, 1985; Stewart 1990; Wald 1984). Similarly I do not give introductions to each of the areas of GR in which CA has been used: I assume the reader already knows about GR or can find the necessary information via the applications papers cited.

## The nature of computer algebra systems

Systems can be classified in various ways, for example by their approach to simplification (Moses 1971; van Hulzen and Calmet 1983) or by their evaluation strategy (Hartley 1996): some such issues, and related design considerations, are discussed here. Interestingly, most of them were already perceived and discussed by the time of the Yun and Stoutemyer (1980) and van Hulzen and Calmet (1983) reviews.

CA systems differ significantly from numerical programs in their structures and strategies. The most basic difference between algebraic and numerical calculations by machine lies in the storage requirements. This leads on to the need for large total memory, problems of intermediate expression swell, issues in the choice of implementation languages, the demand for good simplification routines, and the strong data dependence of execution times. [Execution times for the same programs using the same system on different hardware can also show unexpected performance ratios, possibly due to differing versions of the implementation languages (MacCallum 1989 {Reduce}).]

In a numerical program, the storage required for a particular variable is predictable in advance: double precision floating point numbers in FORTRAN 77 by default used 64 bits, which gave about $10^{19}$ distinct numbers (though their magnitudes could be much larger than that, depending on the division of the 64 bits between exponent and mantissa). Thus the programmer could work out in advance the total storage requirement of a program, and find out whether it would run on a PC or needed a supercomputer. The same applied to estimating computation times.

In CA, this is no longer the case. The algebraic expression which is the value of a variable may be of any length, and even such a simple expression as $2x^3+y^2$, converted to a commonly-used, if old-fashioned, input-output format^9^ as 2*x**3+y**2 and stored as 8-bit ASCII code, would already take more than the 64 bits of double precision FORTRAN 77. This basic difference between symbolic and numerical calculation has a number of side-effects.

The first is that the overall storage requirement tends to be high. Now that typical PCs have 64-bit CPUs and at least 4 Gb RAM, this is less of a problem than in the past. A critical point was reached some years ago when memory became cheap enough to prompt researchers to overcome memory bounds by buying bigger machines rather than spend 6 months thinking of ingenious ways to pack the calculation into available store. As a result systems have tended to become swollen in size and inefficient, even if for routine problems they still run in times and memory sizes acceptable to users.

CA systems are in any case large: under Linux on my PC, Axiom occupies 3.05 Gb of the file store, Maple 2017 2.41 Gb, Mathematica$^{\textregistered }$ 11.2 9.81 Gb, the main “trunk” Reduce directory 1.74 Gb, and Sage-8.0 (built from source) 8.53 Gb. Maxima as a standalone package has a surprisingly small set of download files given Macsyma’s reputation for being large.

It should not be assumed that these figures measure differences in capability or efficiency. CA systems may come with extensive directories of applications, and for the sake of independence from the underlying operating system version, may include operating system software or libraries. In particular the Sage sources for Linux include the versions of compilers and libraries required to build and run Sage. While this avoids the problems given by those other systems and packages that can be difficult to install because they require specific revisions of system programs or libraries which may not be present, it significantly increases the store required (the pre-compiled binaries for Sage are much smaller). Sage also incorporates many other programs, again increasing storage use: see Sect. 3.2.5.

A second is “intermediate expression swell”. As an example, consider the multiplication of 2 univariate polynomials of 10th degree with numerical coefficients, each having 11 terms. If one calculated all possible cross-products of their terms before simplifying, one would obtain 121 terms, while the final answer must be a 20th degree polynomial and thus have at most 21 terms. So in the middle of this calculation one would have 100 more terms than at the end. This has two consequences: one of them is to again boost the overall storage requirement, but the new feature is the importance of using software providing “garbage collection”, where storage used but no longer required is re-allocated for a new use. (See also Padget 1982.)

It is not impossible to implement garbage collection in FORTRAN, but it is not a standard feature, whereas it is standard in Lisp and C. That is why those languages have been the most common bases of CA systems, with the more recent addition of Python.

I shall not try to describe those programming languages in any detail here but just give some references. Winston and Horn (1981) is a good introductory text on Lisp and described the variant Maclisp used in the initial implementation of Macsyma: there are some appendices mentioning other Lisps. Lisp naturally operates on lists, which is how algebraic expressions can be regarded: “Lisp” stands for LISt Processing (though because of the formatting commonly used, with many brackets, it is jokingly said to mean “Lots of Irritating and Superfluous [or Silly] Parentheses”). A Lisp list may itself be composed of lists or of indivisible “atoms”. Lisp has a number of dialects which differ in (e.g.) the way in which macros are expanded.

C (Kernighan and Ritchie 1988) grew from an earlier language B (which in turn developed from BCPL) and was the language in which Unix and its later derivatives were and are written. It is well-adapted for system programming. Many applications nowadays use its object-oriented extension C++ (or the GNU version G++). A number of Lisp systems have been written in C or its extensions, e.g. the CSL used by Reduce.

Python (Ramalho 2015; Stewart 2017) provides object-oriented programming and structured programming paradigms, and has features and extensions supporting further styles: it is used by Cadabra and in Sage.

One can extend a CA system with additional code in the implementation language, but each of the systems described in Sect. 3 also has its own programming language for users. For most of the systems, these are rather similar even though the implementation languages may be quite different.

For example, although one can program Reduce directly in Lisp, it, like Macsyma, provides an ALGOL-like interface. This is internally parsed into the Lisp, and has two modes, algebraic and symbolic (see MacCallum and Wright 1991). Maple has a user language similar to PASCAL. Mathematica$^{\textregistered }$ is somewhat different as it makes idiosyncratic use of non-alphanumeric characters (such as @, #, and |). As an example of the sometimes confusing differences, Mathematica$^{\textregistered }$ and Sage use SHIFT-RETURN to terminate interactive commands, rather than RETURN (often labelled ENTER), while in Maple this is used to mean that the command continues on a new line.

These language choices however acted, and to some extent still act, as an inhibiting factor in the spread of CA. Users are reluctant to learn a new language, and still more so a new programming style. It used to be said by my collaborators in Stockholm that one could learn Lisp programming in 3 weeks unless one had learnt FORTRAN first, in which case one must double the time. In particular, recursive algorithms, which were impossible in FORTRAN, are almost essential in CA as a way to cope with expressions whose length is a priori unknown.

Avoiding intermediate expression swell affects algorithms. For example, when forming a product of three expressions *a*, *b* and *c*, it may be useful, having checked in advance whether any of them simplifies to 0, to multiply (say) *a* and *b* and simplify the result before multiplying by *c*.

The third effect is indeed the need for, and difficulty of, simplification routines. One may want these in order (Fitch 1973; Yun and Stoutemyer 1980) to make expressions smaller (to save on intermediate expression swell), to make them more intelligible to humans, or to check whether an apparently non-zero expression is actually zero. It is a theorem that even apparently elementary classes of simplification problems are not formally decidable (Buchberger and Loos 1983): for example, there is no algorithm guaranteed to simplify to 0 in finite time all expressions which are equivalent to 0 and are expressed by applying $+$, −, $\times$ and $\div$ to rational numbers, *x* and $\pi$, using also $\exp$, $\log$, $\sin$ and the modulus |  | (this result is due to Richardson (1968) and Caviness (1970); see also Fitch (1973)).

Hartley (1996) described simplification as “one of the most confusing and at times irritating areas in CA for the user” and remarked that “The difficulty with ‘simplify’ as it is used in the physics literature is that it is too vague”. Yun and Stoutemyer (1980) listed a very large number of aspects of the problem and the choices involved for both designers and users, and Moses (1971) noted the dependence of some simplification methods on the correctness of number theoretic conjectures.

Simplifications may be of such a character that it is always safe to apply them: for example $0\times x= 0$. More generally one may want to have a “normal” simplifier, which is one that returns 0 for any expression equivalent to 0 (as stated just above, a usable normal simplification algorithm may not exist for some classes of expressions). One may further want a “canonical form” so that equivalent (non-zero) expressions have a unique representation. There are many theorems about simplifiers and the issues also link to problems of “term rewriting”, theorem proving and logic programming.

Frequently, there are simplifications special to the problem, requiring good control of substitutions or rewrite rules, preferably with an interactive interface so that one can inspect the results of trial simplifications readily without over-writing the expression to be simplified and thus perhaps needing to recompute it. In general one needs some human intervention to set and control the pattern matching involved in most simplifications. Thence CA systems only really took off when interactive operating systems became the norm. For some more on simplification see the texts cited above: Caviness (1970), Moses (1971), Fitch (1973), Yun and Stoutemyer (1980), d’Inverno (1980), Buchberger and Loos (1983) and Stoutemyer (2011).

In revisiting simplification issues recently, Stoutemyer (2011) set out 10 “Goals”. Several of these relate to issues about domains of definition: Stoutemyer begins by pointing out that a simplifier may need to know whether a function is real valued, whether its value is necessarily finite or can be infinity, and whether the value is unique (for example, $\sqrt{a^2}$ might be *a* or $-a$). He amusingly defines ‘candid’ and ‘misleading’ expressions, showing examples of the latter which many systems fail to simplify satisfactorily. He also shows that no one canonical form is always good for all purposes. There are too many further interesting points for all of them to be summarized here, among them some of the difficulties system designers have with the range of different results users might want.

There are simplification or substitution problems which may be decidable but for which no algorithm is yet known, for example the problem mentioned above of achieving a shortest expression using “sum-substitution”.

Another comment worth making is that execution times can be very much affected by *when* a substitution is made. If a quantity *Q* has as its value a long expression, and *Q* appears in various steps of a calculation, the best strategy may be not to use the value of *Q* until the final step, carrying *Q* up to then as a formal symbol. This does depend on how the system performs the steps involving *Q*. (See for example Nielsen and Pedersen 1988; Schrüfer 1988; MacCallum 1989 {Reduce}.)

These sorts of issues imply that execution times, and even the feasibility, of particular calculations, are not only data dependent but also dependent on the ways systems deal with the data, i.e. the design choices and available options.

There are some common bases for any reasonably general CA system, for example the implementation of efficient integer arithmetic for arbitrary size integers, and good algorithms for polynomial arithmetic: algorithms for such basic facilities are described in modern texts. Beyond that, there are quite a number of choices which have to be considered when building an algebra system. Many of the design decisions are more computer science than mathematics. Among these are the following.

**1.** **Amount of mathematical knowledge**

This affects the class of problems one can treat. It may sound good to expand that class as far as possible, but each capability, especially if automatically invoked, carries an overhead. Hence special purpose systems, omitting capabilities irrelevant to the class of problems treated, can be more efficient. Areas which have generated special purpose packages include quantum theory and celestial mechanics as well as GR, and there also exist special systems for pure mathematical problem domains such as ring theory or Lie algebras. A subsidiary issue is to decide for what class of inputs the programs should be optimized. This again may favour special purpose systems, like those in Sect. 7.

**2.** **Programming style**

While all the general purpose CA systems described in Sect. 3 seem to use elements of all the main styles, they each emphasize certain aspects more than others. Each of the choices has advantages and disadvantages.

- Reduce and Macsyma are based on a functional programming style. This enables natural expression of the mathematical idea of a function, i.e. a mapping which takes a value in some domain and returns a value in some range. For example, one might define a function *factorial* which acted on an integer *n* and returned the value of *n*!. Such functions can be composed, e.g. one could evaluate *f*(*g*(*x*)) if *f*(*x*) and *g*(*y*) are functions defined for suitable *x* and *y*, and they can be used recursively (e.g. defining  *factorial* using $factorial(n) =n*(factorial(n-1))$).
- Maple makes essential use of hashing techniques and stores quantities in dynamic arrays. There are a number of internal data representations. Simplified expressions are stored only once and assigned a signature which is independent of the order of terms (Char et al. 1983). This design improves efficiency, but the order in which expressions occur in the course of a session may then affect how they are stored and hence, for example, the order in which terms in an output expression appear.
- Mathematica$^{\textregistered }$ makes considerable use of rewrite rules, i.e. has more extensive pattern-matching than other systems. This can make implementation of substitutions, or of rules such as symmetries of tensors, easy for users. An example would be using the identity $e^{i\pi }=-\,1$. The main disadvantages are that if a very large set of rules have been defined, it may take a long time to check all of them to see if they apply, and that the order in which rules are applied may be important but not easy to control.
- Axiom uses object-oriented methods. For example it gives an object-oriented definition of polynomials over an arbitrary ring and of operations on them: they will then inherit whatever operations are defined in the specific ring.

**3.** **Data representation**

The data representation can affect efficiency and the class of optimally-treated problems, and can have an enormous effect on the speed and the possible features. For example,

- A dense representation provides fast manipulation for dense polynomials: here every possible power is assumed to be present, so only a list of coefficients is required. This becomes extremely wasteful for multivariate polynomials with many terms absent; the latter require a sparse representation, in which not only coefficients but also powers are explicitly recorded.
- Polynomials may be stored in a factored or an expanded form; for example $$(1+x)^{100}$$ or $$1+100 x+ 4950x^2\ldots $$ and may be in a distributed or a recursive representation, for example $$1+2x+2y+x^2 +4xy+5y^2$$ or $$ (1+5y^2)+(1+4y)x+x^2$$.
- maple’s tree structure for data may affect its substitution capabilities, making it harder to substitute for $a+b$ in $a+b+c$.
- reduce’s data representation, which by default is a nested list structure, may be the reason for its superior performance on the example of Yamartino and Pavelle (1991).

Some interesting reflections on representations were given by Hearn (1985). For a comparison between data structures see Fateman and Ponder (1989).

Stoutemyer (2011) stresses the impact of the choice of representation on simplifications, and advocates and describes a particular form of representation as a way to achieve the “Goals” he set out for simplification; it is the “recursive partially factored form” that was used in Derive (see Sect. 3.3.2), and was based on the form used in the 1960s package ALTRAN.

**4.** **Choice of algorithms**

CA systems use methods of calculation which can be very different from those a human would typically use in a hand calculation; factorization of polynomials over the integers, and indefinite integration, provide examples. The user unaware of how these calculations are done may be left puzzled by the behaviour of the system and unable to exploit its features fully.

For certain problems a “best” algorithm may not exist, and the CA system will then necessarily perform well on some examples and poorly on others (some systems are provided with more than one algorithm for certain tasks, but in such cases there is often no simple way to decide from the input which algorithm to choose, so even this may not help).

As mentioned above, there are questions for which only heuristics, sometimes unreliable ones, are known, but which users would like the system to deal with; the unfortunate system providers are criticised for incompleteness if they leave such heuristics out, and for incompetence if they put them in. (The most obvious example of such a feature is given by definite integration when contour integrals are required.)

**5.** **Use of datatypes and overloading of operators**

Some systems (e.g. Axiom) use a strong type scheme; then parametrized data types can take advantage of an object-oriented design. A disadvantage of such a scheme is that the system might not accept addition of a polynomial and a truncated power series until one has been converted (“coerced”) to the type of the other.

Hartley (1996) states as an advantage for the user that “most systems are (virtually) type free”. Most systems also, relatedly, “overload” the operators of some common operations, for example multiplication, so that a given command can produce different operations for different types of data: multiplication of two integers is not the same as multiplication of two matrices. (This can sometimes be confusing if the symbols presented to the user are identical even though they stand for different processes.) Overloading is typically achieved by defining a function which first tests the types of the data and then calls the appropriate subroutine. To avoid overloading one may define different functions for various data types and require the user to choose the right one or, in a higher-level design, use inheritance properties in an object-oriented system.

**6.** **Evaluation of chains of assignments**

One aspect of evaluation strategy is the handling of chains of assignments (another is mentioned in point 8 below). Suppose (following Hartley 1996) we have made the assignments

2 $$\begin{aligned} a\leftarrow & {} b +1 \end{aligned}$$

3 $$\begin{aligned} b\leftarrow & {} 0 \end{aligned}$$

4 $$\begin{aligned} c\leftarrow & {} a \end{aligned}$$

and we ask the system to evaluate *c*. If it makes a simple evaluation the result is *a*. If the program automatically follows the assignments one step the result is $b+1$. This is, or used to be (I have not checked that the same applies to the latest versions) done in Axiom, and Macsyma. Maple is (or was) single-step inside procedures but *n*-step or indefinitely many at the interactive level—which could trip programmers up when debugging. If the program automatically follows the assignments as many steps as it can (which Reduce does), the result is 1. Mathematica$^{\textregistered }$ was able to treat an expression as one step or in an indefinite number of steps (I have not checked if this is still true).

One also has to beware of infinite loops if the system can make indefinitely many steps through the assignments, e.g. asking for the value of *c* after

5 $$\begin{aligned} a\leftarrow & {} b +1 \end{aligned}$$

6 $$\begin{aligned} b\leftarrow & {} a \end{aligned}$$

7 $$\begin{aligned} c\leftarrow & {} b \end{aligned}$$

would create a never-ending loop (which many systems would detect, returning an error after 2 or 3 steps).

Another aspect is that one wants to avoid repeated evaluations of the same quantity, which can easily arise when dealing with recursive functions. An example is that of defining the Legendre polynomials recursively via the recurrence

8 $$\begin{aligned} n P_n (x) = (2n-1)xP_{n-1}(x) - (n-1)P_{n-2}(x) \end{aligned}$$

(with $P_0=1$, $P_1=x$). Done incautiously, one would end up evaluating $P_{n-2}$ twice and so on (cf. Fulling 1991). So in a “lazy evaluation” strategy, where a value is computed only when it is required, and otherwise only the way to compute it is stored (this is used in calculating geodesics in GRWorkbench and in the power series packages of Reduce and Sheep), storing those values that have been computed may also improve efficiency.

A further issue is that the result of evaluations may depend on the context in which the values were assigned, i.e. the way in which bindings of values to variables are handled (some traps for the unwary are noted on pages 259–260 of Yun and Stoutemyer 1980).

**7.** **Control philosophy**

Moses (1971) characterized systems’ simplification processes as “radical” if the output has to be in a canonical form, “new left” if the user is allowed some control over the expansion, “conservative” if only trivial simplifications, such as $x+0 \rightarrow x$, are done automatically, and “catholic” if the user can choose which is to be done. An example of the resulting differences is that in Maple the function normal sometimes has to be called to do what Reduce would do automatically. Stoutemyer (2011) discusses what the design of systems’ defaults (i.e. with no call such as to normal) should cover, and singled out those systems, mostly for educational use, in which individual steps of simplification are or can be followed.

**8.** **Further issues**

There are some important additional issues requiring choices. One is the evaluation strategy applied when a variable which has been assigned a value is given as an argument to a function. There are quite a few approaches but the two most common are “call by value” and “call by reference” (others are “call by name”, ”call by sharing”, etc.). In call-by-value, the function evaluates its argument and uses the value found, usually copying it: the variable’s value, which might be used elsewhere, remains unaltered. In call-by-reference, the function uses a reference to where the value is held, so that it could assign the variable a new value which would then be used elsewhere. FORTRAN II used call by reference. Most CA systems use call by value as default, though they may provide call by reference as well.

Related to this is the question of the scope within which a variable has a certain value. Global variables have the same value in all places, so if the value is changed somewhere that value will be returned wherever the variable is subsequently evaluated. Local variables may have one value within a procedure and a different value elsewhere, and changes within a function or procedure do not propagate elsewhere. The use of “packages” or “closures” provides more flexible ways to manage the scope of variables’ values.

Another issue is the order in which a function with several arguments evaluates them (e.g. left to right, right to left, ...).

One may note that an important efficiency aspect may be whether a program is run interpreted (i.e. executes instructions without compiling them into the machine’s internal codes) or compiled. The latter will usually be much faster, but not all CA systems’ interface languages offer compilers. As far as I can tell from their documentation, Maple can compile a limited subset of procedures, via C, and Mathematica$^{\textregistered }$ can compile functions assuming or being given the argument type: it appears neither can compile a whole module. If a system offers a compiler, a good strategy in development can be to run code interpreted until its correctness has been tested, and then compile it. This is possible, for example, in Reduce and Sheep, and most of the modules in the standard distributions of those systems are compiled when the systems are built.

Thirty years ago it was easy to list many aspects in which the current systems could be improved. One might have listed: more powerful portable systems, available on devices that would fit in a pocket; improved symbolic-numeric interfaces; embedded systems; graphical output; easier user input including symbol menus, pen pad input, and two-dimensional input; and improved help systems. These have virtually all come to pass, though not all in any one system.

Fuller explanations of some of these aspects (with some consideration of the systems’ uses in GR) are given (e.g.) in reviews by me (MacCallum 1996, 2000) and Hartley (1996): although these are out of date in their descriptions of systems, the set of design options has been rather stable (Grabmeier et al. 2003).

## General purpose systems

There is no clear definition of a general purpose system. Here I shall consider as general-purpose systems those which can carry out the mathematical operations of the calculus and algebra common in mathematical methods courses and mathematical physics. This means I will not discuss the rather general systems that are primarily aimed at pure or discrete mathematics such as MAGMA or GAP.

While the subset of facilities common to all the general purpose systems (for this paragraph, including those such as GAP) may be small, a general purpose system will have a rather large set of capabilities. Past attempts at system comparisons have constructed tables showing which systems offered which features, but such a table would now be so extensive it would be very hard to construct reliably and be unreasonably long. Such a table would anyway not help one to define which systems are general-purpose and which are not.

It should be noted that most modern general purpose CA systems offer numerical and graphical capabilities as well as algebraic computation, and have graphical user interfaces, following the introduction of such features in Mathematica$^{\textregistered }$ (see Sect. 3.1.3).

All general purpose systems implement utilities such as factorization of polynomials over the integers, integration of rational functions, and so on, the algorithms for which are discussed in texts such as those cited earlier, e.g. Geddes et al. (1992) and von zur Gathen and Gerhard (2013). A number of more recently-developed algorithms, or extensions of the standard ones, may not be, or may not be fully, implemented in all systems. Examples are van Hoeij’s knapsack method for factorization of polynomials over the integers (van Hoeij 2002), Wu’s characteristic sets method for solving systems of polynomial equations (summarized in Wu 2001), and recent methods for differential equations (see, e.g., papers in MacCallum and Mikhailov 2009).

The systems also offer interactive command-line interfaces and windowing interfaces, the latter generally by using the operating system’s native windowing systems (e.g. the eponymous Windows or, for example, X Windows). The command line interfaces, though they may be considered superseded for interactive use, are very useful for running sets of commands in a batch mode, which may be less convenient to do using, for example, Maple ’s worksheets or Mathematica$^{\textregistered }$ ’s notebooks.

The output routines now at least offer a two-dimensional text layout in which indices are raised and lowered appropriately, and most if not all systems make fully formatted output, looking like printed mathematics, available, in many cases through the medium of  . Being able to save output in  form has obvious advantages in preparing publications, although that form would not be suitable for feeding into subsequent steps of a calculation; a trivial example of the difference is that one may want to see ${R^i}_{jkl}$ printed in the output or shown on screen, but for further calculations one needs to know whether the *jkl* is three indices or one index with a three-character name. So in order to carry out further operations on the results, one usually needs them in the relevant input format. In many cases the input formats are either like FORTRAN arithmetic expressions or use a prefix notation.

Because the various general purpose systems, as well as more special systems, offer different facilities, it could be useful to pass problems from one to another and use the best features of two or more systems. (To some extent this is a motivation for Sage.) To do so requires a common semantics and syntax to provide intercommunication between the systems; this aim is not met by, for example, Presentation MathML (see [C77] for fuller information about MATHML and its variants), which is concerned with visual rendering, but may be met by the MathML variants which cover semantics.

Such intercommunication can be achieved for specific cases by do-it-yourself methods, for example by using perl scripts, but that is clearly of limited utility. In principle OpenMath [C78], which aims to capture semantics as well, offers considerable prospects but it has not developed far enough yet to provide a universal solution to the difficulty; however, proof of concept work has succeeded (Berth et al. 2000). An alternative approach is code-sharing via pseudocode, which has been validated in examples such as work on linear ordinary differential operators (Wright 1995).

Most of the packages in Sects. 6 and 7 developed separately from one another although better and more comprehensive packages might have been developed had individuals and groups combined their efforts. Some of them do make use of others, for example combining Mathematica$^{\textregistered }$ and FORM (Cyrol et al. 2017), but intercommunication between them, such as Pollney’s linking of Sheep and GRTensorII (d’Inverno 1998) has not been much used.

As already mentioned, the earliest of the current major systems, with origins in the 1960s, were Macsyma and Reduce, both Lisp-based. Work on muMATH, designed for the early PCs, began in the 1970s. IBM had started to develop a system Scratchpad in the 1960s but this was replaced by Scratchpad II in 1977; that became Axiom, which is still available.

The 1980s brought the two most successful commercial systems, both C-based, Maple and Mathematica$^{\textregistered }$, which have become powerful pieces of software with many add-on packages for special purposes. These are also as far as I know the only two independent (i.e. available other than as part of a software suite) systems that are still commercial. The even more recent system MuPAD, developed at the University of Paderborn, was eventually sold to MathWorks.

Finally the open source system Sage, which incorporates many other pre-existing programs for a range of mathematical sub-disciplines including the Maxima derivative of Macsyma, has become very popular and offers a very wide range of capabilities. The systems mentioned in the last three paragraphs, and some others, are described more fully in Sects. 3.1.1, 3.1.2 and 3.3.3.

I believe all the current general purpose systems are available under recent versions of Windows, Apple operating systems and Linux. (I have heard that although Sage is not directly available for Windows it runs fine within a virtual machine.) I do not know which are currently available for smartphones and other devices (as a past example, Derive was available on the calculators made by Texas Instruments (TI), and TI later developed their own specialized system; currently Xcas [C16] is available on some devices). Some systems provided online versions usable remotely, i.e. where the actual calculation is done at a remote host site (see e.g. Ishak et al. 1999 for an early example): Wolfram|Alpha now provides the most extensive such service (see Sect. 3.1.3). (In the early days, when only specific machines could support CA systems, people often accessed those machines remotely via modems. However, this could not be done with the speed and immediacy modern links provide.)

For detailed documentation of almost all systems and packages the accompanying manuals are the primary source of information. These are typically circulated in electronic form, whether on CDs or DVDs or via the Internet, and are thus obtainable via the URLs listed in the appendix. I have therefore not listed them in the bibliography. The more recent systems also have rather extensive internal or online help facilities. Some introductory texts are listed below, as are references to the extensive bibliographies available for the main systems. Nelson Beebe maintains bibliographies [C61] for the following systems: AXIOM and Scratchpad, MACSYMA and VAXIMA, Maple, Mathematica$^{\textregistered }$, MATLAB, and Reduce. When there has been a publication describing a package, online citation information may help one find applications of that package.

### The principal general purpose systems used in GR

#### Macsyma (and Maxima)

Macsyma was developed at MIT from 1968 to 1982 as part of the MAC project (which also led to the well-known Emacs editors, and hence GNU and Linux) funded by DARPA (the Defence Advanced Research Projects Agency, whose networking project was the basis of the internet). It has been implemented then and since in a considerable number of different Lisp systems. The MIT team included many pioneers in the field and developed many of the fundamental algorithms of CA, so the impact of the work done then is still substantial. For a history of that period and later developments see Moses (2012).

Macsyma’s ownership history is rather convoluted. (Note: I am not confident that the following is entirely accurate, but the same may well be true of the longer version available on Wikipedia [C60]). The rights to MIT’s Macsyma passed in 1982 to Symbolics Inc, a company making Lisp machines, hardware first developed in the early 1970s that was specially designed to run Lisp programs. Macsyma was further developed by Symbolics and by Macsyma Inc, which purchased the rights from Symbolics Inc in 1992; its capabilities were very competitive with other systems at that time. In 1985 there were 415 papers listed in the Macsyma bibliography maintained by Symbolics. However, commercial pressures and the unwillingness of investors to put in necessary capital eventually led to the sale of Macsyma Inc to the same company that had bought Symbolics Inc. The rights to this version remain with Symbolics, now a private company: its website [C1] still lists Macsyma 2.4 as available for Windows PCs with operating system versions up to the now discontinued XP. Some later promising attempts towards reviving this version of Macsyma came to naught in competition with funding for more abstract pure mathematics (Richard Petti, private communication).

Because the MAC project had received US Government funds, the rules governing US public funding meant that the US Department of Energy retained the rights to a version, DoE-Macsyma (Carrette and Harten 1985). A licence was also granted to the University of California Berkeley and Macsyma was eventually implemented using their *Franz Lisp* (later Allegro CL). At one time this version was referred to as Vaxima, because it was available in particular for DEC VAX machines.

DoE-Macsyma was for some years maintained by Paradigm Associates. It is this version which has become Maxima [C2], which was released in 1998 under GPL (GNU General Public License). It does not have all the facilities that were added after Macsyma and DOE-Macsyma started to develop separately, but is under active development. It has a number of extensions and interfaces with other systems and is available for the main operating systems. It is written (now) in Common Lisp; the website lists the GNU Clisp implementation as a preferred base. Maxima is a component of Sage, which provides as the Lisp base ECL (Embeddable Common Lisp).

For completeness, as papers may reference them, I note that there were earlier versions of Macsyma for Macintosh computers (aljabr from Fort Pond Research, and paramacs from Paradigm Associates) both being licensed from DoE-Macsyma.

Davenport et al. (1993) begins with an introduction to Macsyma, and Maxima has a very extensive manual.

#### Maple

A group at the University of Waterloo, Canada, began to develop Maple [C3] (Char et al. 1983) in late 1980 and it was first released soon afterwards. One aim was to have a system that would be small and fast enough for whole classes of students to use simultaneously on the time-shared machines of the day (Macsyma was not suitable for that, being only available on one machine not readily accessible under the poor network connectivity of the time, and requiring too much time and store for time-shared use in teaching: it was at one time claimed to be the largest Lisp program written).

A second aim was portability, so Maple was written in languages in the BCPL family (initially Maple was written in the common subset of B and C, and a custom macro processor, Margay, was used to specialize to one of the two; later only C was used). Maple has undergone substantial development in its interfaces and libraries, and has also changed ownership. The graphical user interface was first released with Maple V. Versions continued to be numbered up to Maple 18, and then changed to an annual label: the current version is Maple 2017. Maple ’s small kernel and its large library, with good interfaces, have led to its wide use, especially in research and higher-level education.

As use of Maple grew, a spin-off company, Waterloo Maple, was formed, and the rights are now with Maplesoft (see [C3]). Early versions distributed the commented sources of the library code (though not the kernel C code); that has long since ceased to be the case, although one can see the uncommented code for a function by using the help function within Maple.

There are many books on Maple, for example Wright (2002) and Heck (2003), some now out of date in that they refer to rather old versions. For a fuller bibliography of Maple books see [C63].

#### Mathematica$^{\textregistered }$

Stephen Wolfram’s Mathematica$^{\textregistered }$ [C4] replaced his earlier SMP (see Sect. 3.3.3) and was first released in 1988. Development had started in 1986. It was a fully commercial CA system from the start. As of February 2018 it was at version 11.2. It has been very successful and is perhaps the CA system best known to physicists, possibly because its originator, Wolfram, is also a physicist. It has been jokingly called the IBM of CA (referring to the time when “nobody got fired for recommending buying from IBM”).

On its first release in 1988, it was the only system offering a comprehensive computational environment integrating numerical and graphical facilities with algebraic ones, and had a unique user interface, with “notebooks”: it was (and is) thus not only a CA system but a more general system for mathematics. In these respects it outpaced other systems, whose progress in those areas, while not non-existent, had been up to then been leisurely. (Other systems soon followed suit in providing such integrated graphical, numerical and interface features, and by now the other main systems have generally caught up.) Mathematica$^{\textregistered }$ has become a large system with many applications-oriented features. My impression is that its advertising prompted a tendency for all systems to overstate their powers in the hunt for sales: this is regrettable but was probably inevitable.

Mathematica$^{\textregistered }$ is based on C rather than Lisp. It has been claimed this makes it more efficient, but that may be more an issue of implementation details than of underlying language (Fateman and Hayden 1996). In its early history there were some amusing bugs (see Fateman 1989, 1992), but these are of course long gone.

The standard source on Mathematica$^{\textregistered }$ was Wolfram (2003), but rather than continue to produce new editions of that book as the amount of documentation and the number of the packages within the system has grown, users are now referred to the Wolfram Documentation Center [C65] for information. I have used some introductory texts such as Crandall (1991), but do not know the available books well enough to pick out particular ones among them: for a full booklist see [C64].

The Wolfram Language, which “grew out of Mathematica” is the implementation language of Wolfram|Alpha [C5], which provides an online service “doing dynamic computations based on a vast collection of built-in data, algorithms and methods”. It aims “to make all systematic knowledge immediately computable and accessible to everyone”.^10^

#### Reduce

Reduce is a highly portable system available on a wide range of machines and was probably at one time the most widely used in a geographical sense. It was initially written by A.C. (Tony) Hearn for use in scattering problems in quantum theory. It is written in Rlisp (Marti 1993), itself written in Standard Lisp (Marti et al. 1978, distributed with Reduce). Its standard test inputs include a straightforward general relativity program due to Barton and Fitch which computes the Einstein tensor from a metric.

Reduce was for a while a commercial product of the Rand Corporation, but it is now available free and can be downloaded via its website [C6]. The Reduce distribution offers two Standard Lisps, PSL (Portable Standard Lisp) and CSL (Codemist Standard Lisp) and users may build from either (or both) depending on their hardware and operating system. The last release with a version number was Reduce 3.8 but the system has been developed since. The present sources are organized under the subversion program svn and change frequently (as of February 2018, my copy was for subversion number 4441). Unusually, the source code repository provides, alongside the current “trunk” version, a “historical” archive of some preceding versions’ source code (not necessarily possible to rebuild from).

Being free, like Maxima, Reduce lacks the salespeople and glossy brochures of the main commercial systems, but it is an extensive system still under active development which distributes its complete source code (a description which also fits Maxima and Sage). For that reason it has been especially popular with algorithm developers, so that Reduce still has in some areas (in my view) the best current auxiliary packages. That may also be why a number of CA packages for GR were or are built on Reduce.

The introduction to Reduce of which I was a co-author (MacCallum and Wright 1991) is still useful although it was written before the switch from upper to lower case as default. For other books see the ‘Books’ tab at [C6]: some such as Grozin (1997) are aimed at particular application areas, but none I know of cover the same ground as MacCallum and Wright . For references, consult Nelson Beebe’s bibliographies [C61] and the ‘Bibliography’ tab at [C6].

### Other current systems

Here I briefly introduce other general purpose systems which could be, but to the best of my knowledge have not been, or not yet been, used to support CA in GR.

#### Axiom

A group at IBM led by the late Richard Jenks created a very high level design for a system known in development as Scratchpad II. The project started in 1980 as a follow-on from the earlier Scratchpad, which had been written in FORTRAN and developed since 1965. Scratchpad II was intended to exploit high-level mathematical concepts to produce a clean and powerful design, having a very abstract approach to datatypes, based on category theory and the object-oriented programming approach, which it embodies.

In 1990 it was sold to NAG (the Numerical Algorithms Group) and renamed Axiom. Axiom (Jenks and Sutor 1992) is a large and powerful system. NAG ported it to Codemist Common Lisp, but stopped selling it in 2002 and eventually released it to be distributed as free software. (Codemist also created Codemist Standard Lisp (CSL), now one of the bases for Reduce.) There are now three freely available descendants, Axiom [C8], FriCAS [C9] and Open Axiom [C10], due to disagreements between maintainers and developers about various issues. Axiom is currently supported by CAISS, the Center for Algorithms and Interactive Scientific Software, a joint effort of the Computer Science and Mathematics Departments of The City College of New York.

Axiom had a lead in some features, e.g. indefinite integration. A (cross-)compiler, Aldor, conceived as an extension language for Axiom and originally named A#, which can accept and output several formats and has libraries embodying the Axiom domain and inheritance properties (Watt et al. 1994; Watt 2003), has also now become freely available [C11]. It may be of interest for symbolic calculation in GR because of its high-level features. However, there are as far as I know no gravity or differential geometry programs yet in Axiom or Aldor.

In my view, Axiom deserved to be much more widely used than it was, but the present divergence into multiple descendant systems makes that an unlikely future.

#### FORM

FORM [C12] (Kuipers et al. 2013) was developed, initially by Jos Vermaseren, to handle very large expressions efficiently, optimizing the processing speed and making use of disk storage when necessary, rather than “mimic the way humans work with formulas as much as possible” which its website says other CA systems do. Its applications have been mainly in theoretical particle or high-energy physics, for example in evaluation of Feynman diagrams. Initially written in FORTRAN 77 it was later rewritten in C and eventually made open source. There are now parallelized and multi-threaded versions. Its potential use in GR is principally in quantum GR.

#### MuPAD

MuPAD was developed at the University of Paderborn (Gerhard et al. 2000). It was eventually purchased by MathWorks. It is no longer sold as a separate product but is included in the Symbolic Math Toolbox add-on for MATLAB, which, from the company’s website [C13], appears to mean it can still be used as a CA system. I am not aware of any geometric or GR packages written in MuPAD.

#### Redberry

Redberry is described in Bolotin and Poslavsky (2013) as “an open source computer algebra system with native support of tensorial expressions. It provides basic computer algebra tools (algebraic manipulations, substitutions, basic simplifications etc.) which are aware of specific features of indexed expressions: contractions of indices, permutational symmetries, multiple index types etc. Redberry supports conventional  -style input notation for tensorial expressions. The high energy physics package includes tools for Feynman diagrams calculation: Dirac and SU(N) algebra, Levi-Civita simplifications and tools for one-loop calculations in quantum field theory. Redberry is written in Java 7 and provides convenient Groovy-based user interface inside the high-level general purpose programming language environment.” From this description it may deserve to be placed with standalone GR packages rather than general purpose systems. Redberry is available at [C14].

#### Sage

Sage [C7] is a very extensive system for mathematical work which combines fresh code with a number of pre-existing packages. The current list of included packages has 90 entries, among them Maxima (see above), Xcas (see below), the statistical package R, NumPy, SciPy and Sympy (programs for numerical, scientific and symbolic manipulations in Python), and important packages for pure mathematics. Its main implementation language is Python, and its user interface language is similar in character. For speed it also uses the superset of Python, Cython [C70], which can produce compiled C and link with C++.

As already mentioned, it has the commendable feature that the distribution set of Linux sources includes all the packages on which it depends, so that it is entirely self-contained.

There is a differential geometric package for Sage, distributed with the system. It is called SageManifolds and has been developed by a number of authors led by Eric Gourgoulhon (Gourgoulhon et al. 2015) [C52]: see also Birkandan et al. (2017). It uses the iPython interface. There are or were also tensorial packages for other components of Sage: TensorA [C53] for R, written by K. Gerald van den Boogaart, which appears to have been last updated in 2006; diffgeom [C54], defining rather general classes for manifolds; Tensor [C55], which uses array and matrix methods, for Sympy; and GraviPy [C75] which uses iPython.

#### SymbolicC++

SymbolicC++ [C15] (Hardy et al. 2008) “uses C++ and object-oriented programming to develop a computer algebra system.” It “introduces, amongst others, the Symbolic class which is used for all symbolic computation. The Symbolic class provides almost all of the features required for symbolic computation including symbolic terms, substitution, non-commutative multiplication and vectors and matrices.” The system includes facilities for Clifford algebras and Gröbner bases.

One may also note the C++ library described in Limache and Rojas Fredini (2008), but I am not aware of any package based on it.

#### Xcas

Xcas [C16] is a user interface to Giac, a “free, basic Computer Algebra System”. These are open-source projects developed by Bernard Parisse et al. It is based on experiences gained with Parisse’s former project Erable. It can be used “directly inside software written in C++” and has been utilized in a number of mobile devices (e.g. HP Prime, TI Nspire) and pocket systems. It has compatibility modes with Maple and MuPAD, interfaces with a number of well-known libraries, can be used from LaTeX, and there is or was an OpenOffice (now LibreOffice) plugin enabling its use within spreadsheets etc. A version is included with Sage (see Sect. 3.2.5).

### Earlier systems

Here I briefly cite sources for information on general purpose systems which have been used for CA in GR but are, or appear to be, no longer available or maintained.

#### FORMAC

FORMAC was a list-processing extension initially of FORTRAN and later of PL/1 (Sammet 1993). It was revised significantly from 1973 to 1977 (Bahr 1973, 1977; Yun and Stoutemyer 1980). Before 1980, many packages using it in GR were written. It was still available in 1987.

#### muMATH

muMATH (Wooff and Hodgkinson 1987) was based on muLISP and provided a system for CP/M and later MS-DOS microcomputers (before Windows was introduced). Its lead author, David Stoutemyer, designed it to be usable within the small memory of those now obsolete machines (early IBM PCs had 640K of memory). As hardware and operating systems improved, the original system was replaced by Derive, as used in TI calculators (mentioned above). Derive was discontinued in 2007 in favour of a newer TI CA system (see Stoutemyer 2011). MuMATH itself was rewritten for the Atari ST and TT with 32-bit address pointers, allowing a much larger data space, and with a windowing interface: this version, whose manual was as far as I know available only in German, was called riemann.

#### SMP

SMP (Symbolic Manipulation Program: the acronym is amusing to UK users brought up in the era when the School Mathematics Project, a scheme to modernize the high school mathematics syllabus [C79] was being introduced) preceded Mathematica$^{\textregistered }$ as a product of Stephen Wolfram and colleagues. Design work started in 1979 and it was sold as a commercial product from 1981 to 1988. Some claims made for this system (Wolfram 1985) were considered unjustified by other researchers (Fateman 1985; Davenport et al. 1985; Monagan et al. 1986). Experience with SMP presumably informed the design of Mathematica$^{\textregistered }$.

## Calculations in gravity research

There are two main types of algebraic calculation in GR. One is the calculation of general expressions in indexed objects such as the equations defining the Levi-Civita connection and Riemann tensor in coordinate components for a spacetime in general relativity, i.e.

9 $$\begin{aligned} {\varGamma ^i}_{jk}:= & {} {\frac{1}{2}}g^{il}\{g_{j\ell ,k}+g_{k\ell ,j}-g_{jk,\ell }\} =: {\frac{1}{2}}g^{il}[jk,\ell ] \end{aligned}$$

10 $$\begin{aligned} {R^i}_{jk\ell }= & {} {\varGamma ^i}_{j\ell ,k}-{\varGamma ^i}_{jk,\ell }+{\varGamma ^i}_{mk}{\varGamma ^m}_{j\ell }- {\varGamma ^i}_{m\ell }{\varGamma ^m}_{jk}, \end{aligned}$$

(here $=:$ means that the left side *defines* the object on the right, and similarly for $:=$). The other is the calculation of one or more values for specific indices, typically components of tensors, e.g. the component ${R^1}_{234}$ of (10) for given coordinates and metric. These will be referred to respectively as indicial and component calculations.

In indicial calculations, the indices are in effect “abstract indices” (Penrose 1968; Ashtekar et al. 1982; Penrose and Rindler 1984): the set of possible values need not be specified. The indices’ nature (coordinate, orthonormal or null frames, spinorial, etc) generally does not affect packages’ ability to compute with them, though there may be special simplifications to apply, and some packages allow multiple types of index to be treated simultaneously (thus including the ‘generalized tensors’ of Ashtekar et al. 1982).

There are two very useful crossovers between indicial and component calculations which CA packages may provide: (i) a package may provide a facility for generating code for evaluation of components from the indicial form, such as the “tensor compiler” (Hörnfeldt 1976, 1977, 1979) which was used in Holmes et al. (1990), and the ic_convert command in Maxima’s itensor module (see also MathGR in Sect. 6.3); and (ii) a package may allow a component to be expanded in terms of symbolic components for other objects. As an example of the latter, here is an expanded component of (9) (using Sheep where the object ${\varGamma ^i}_{jk}$ is called GAM) for a metric with symbolic entries dependent on all coordinates, here numbered 0–3,
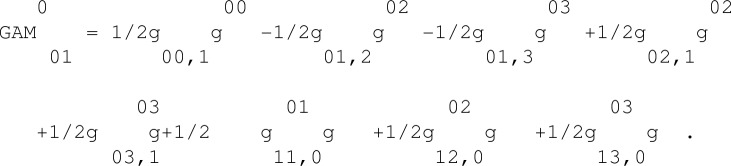


Let us first consider component calculations. Introductory texts on general relativity mostly start with the coordinate component formulae. Direct substitution of values into formulae such as that immediately above can be tedious; to evaluate, for example, ${R^1}_{234}$ from (10) one has to repeatedly look up values of the ${\varGamma ^i}_{jk}$. Computers handle such table lookup much better than humans.

In hand (or “pencil and paper”) calculation a more convenient method than directly using (9) is to note that the geodesic equations

11 $$\begin{aligned} \ddot{x}^i = {\varGamma ^i}_{jk}\dot{x}^j\dot{x}^k, \end{aligned}$$

where the dot means the derivative with respect to an affine parameter, are the Euler–Lagrange equations of the Lagrangian

12 $$\begin{aligned} L = g_{ij} \dot{x}^i\dot{x}^j, \end{aligned}$$

calculate those, and read off the coefficients from (11).

To go from (9) to (10) an efficient hand calculation method is to use differential forms, the connection form and Riemann curvature form for a coordinate basis being

13 $$\begin{aligned} {\varvec{\varGamma }}^a{}_b:= & {} {\varGamma ^a}_{bc} {\mathrm{d}}x^c, \end{aligned}$$

14 $$\begin{aligned} {\varvec{\varTheta }}^a{}_b:= & {} {\mathrm{d}}{\varvec{\varGamma }}^a{}_b+{\varvec{\varGamma }}^a{}_c\wedge {\varvec{\varGamma }}^c{}_b = {\frac{1}{2}}R^a{}_{bcd}{\mathrm{d}}x^c\wedge {\mathrm{d}}x^d. \end{aligned}$$

(For those unfamiliar with differential form methods, Stephani et al. 2003, Chapt. 2, provides a concise introduction.)

One may choose a more general basis of vectors than the coordinate one. Such a choice may be made because components in the new basis are more readily interpretable physically, for example the energy-momentum of a fluid in an orthonormal basis whose timelike unit vector is aligned with an observer’s motion has components interpretable as the energy, fluxes, pressure and stresses the observer would measure. Or it may be that the choice simplifies calculations or facilitates study of conditions applied to the problem, for example in studying algebraically special solutions of the Einstein equations (Stephani et al. 2003, Part III).

In four dimensions a basis of vectors at each point (in general, not a coordinate basis) is called a tetrad or vierbein. Tetrads used are typically chosen to have fixed scalar products between their basis vectors, but this is not always the case. For fixed scalar products the principal choices are orthonormal or Lorentz tetrads and complex null or Newman and Penrose (1962) tetrads.

An orthonormal tetrad $\{{\varvec{E}}_a\}$ consists of three spacelike vectors ${\varvec{E}}_\alpha , \alpha =1\ldots 3,$ and one timelike vector ${\varvec{E}}_4$, such that

15 $$\begin{aligned} {\varvec{E}}_{\alpha }\cdot {\varvec{E}}_{\beta }=\delta _{\alpha \beta }, \qquad {\varvec{E}}_{4}\cdot {\varvec{E}}_{4} =-\,1, \qquad {\varvec{E}}_{\alpha }\cdot {\varvec{E}}_{4} =0, \end{aligned}$$

where Greek indices run from 1 to 3 and the dot denotes a scalar product. The most widely-used notation for orthonormal tetrads is that due to Ellis (1964, 1967) as set out in MacCallum (1973). Variants on this have appeared subsequently (Ellis et al. 2012).

A complex null or Newman–Penrose tetrad $\{{\varvec{e}}_a\}$ consists of two real null vectors ${\varvec{k}}$, ${\varvec{l}}$ and two complex conjugate null vectors ${\varvec{m}}$, ${\varvec{\overline{m}}}$:

16 $$\begin{aligned} \{{\varvec{e}}_a\}= & {} \left( {\varvec{m}},{\varvec{\overline{m}}},{\varvec{l}},{\varvec{k}}\right) , \nonumber \\ g_{ab}= & {} 2m_{(a}\overline{m}_{b)}-2k_{(a}l_{b)}=\left( \begin{array}{r@{\quad }r@{\quad }r@{\quad }r} 0 &{} 1 &{} 0 &{} 0 \\ 1 &{} 0 &{} 0 &{} 0 \\ 0 &{} 0 &{} 0 &{} -\,1 \\ 0 &{} 0 &{} -\,1 &{} 0 \end{array} \right) . \end{aligned}$$

Thus the scalar products of the tetrad vectors vanish apart from

17 $$\begin{aligned} k^al_a=-\,1,\qquad m^a\overline{m}_a=1. \end{aligned}$$

Note that the spacetime remains real (i.e. modelled on $\mathbb {R}^4$) although it is described using complex quantities. Sometimes ${\varvec{m}}$ and ${\varvec{\overline{m}}}$ are replaced by their real and imaginary parts ${\frac{1}{2}}({\varvec{m}}+{\varvec{\overline{m}}})$ and ${\frac{1}{2}}({\varvec{m}}-{\varvec{\overline{m}}})/\mathrm{i}$ in a “half-null” tetrad, so that objects can be explicitly real.

The commutator $[{\varvec{u}},{\varvec{v}}]$ of two vector fields ${\varvec{u}}$ and ${\varvec{v}}$ is defined by $[{\varvec{u}},{\varvec{v}}](f):= {\varvec{u}}({\varvec{v}}(f))-{\varvec{v}}({\varvec{u}}(f))$ for any adequately differentiable function *f*. For any basis $\{{\varvec{e}}_a, a=1\ldots 4\}$, the commutators

18 $$\begin{aligned}{}[{\varvec{e}}_a,{\varvec{e}}_b]=D^c{}_{ab}{\varvec{e}}_c,\qquad D^c{}_{ab}=-D^c{}_{ba}, \end{aligned}$$

define the commutator coefficients $D^c{}_{ab}$. Then the Levi-Civita connection is given by

19 $$\begin{aligned} \varGamma _{abc}= {\frac{1}{2}}(g_{ab|c}+g_{ac|b}-g_{bc|a}+D_{cab}+D_{bac}-D_{abc}),\;\;D_{abc}:= g_{ad}D^d{}_{bc}, \end{aligned}$$

where $g_{ab}= {\varvec{e}}_{a}\cdot {\varvec{e}}_{b}$, the dot again denotes a scalar product, and the vertical bars denote directional derivatives. If we denote the one-form basis dual to the tetrad $\{{\varvec{e}}_a\}$ by $\{{\varvec{\omega }}^c\}$, we can replace each ${\mathrm{d}}x^c$ in (13) and (14) by ${\varvec{\omega }}^c$ to get formulae for the tetrad components of the connection and Riemann curvature forms. For a tetrad basis with fixed scalar products we have

20 $$\begin{aligned} {\mathrm{d}}{\varvec{\omega }}^a=-{\varvec{\varGamma }}^a{}_b\wedge {\varvec{\omega }}^b. \end{aligned}$$

In the Newman–Penrose formalism, each commutator coefficient $D^c{}_{ab}$ is assigned a single-letter Greek alphabet name, and the curvature, Bianchi identities, etc are then calculated using that notation, which is complemented by a notation for curvature components taking advantage of the symmetries of the tensors. The full sets of equations are written out in several references, e.g. the original NP paper; Pirani (1965), Penrose and Rindler (1984), Stewart (1990); and Chapters 3 and 7 of Stephani et al. (2003). (The set in that last reference were checked using the Sheep module NPEQNS with suitable input data.)

Tetrad methods with well-chosen tetrads, expressed in the differential form notation, provide very efficient and reliable means for hand calculations of components, and I normally use them. From experience the same remark about efficiency often applies to CA calculations (Campbell and Wainwright 1977; MacCallum 1989 {CAMAL, Reduce, Sheep}): however, experiment shows that the critical aspect may be the simplifications required or used rather than the choice of basis (Pollney et al. 1996 {GRtensorII}). One drawback of coordinate calculations is that they may obscure invariant properties and so hamper physical study of the metrics under discussion.

The references for the NP formalism cover the closely related two-component spinor formalism, also named NP, which provides a very compact and elegant framework for numerous calculations in general relativity. The underlying mathematical fact is that the (connected) group $SL(2,\mathbb {C})$ of linear transformations in two complex dimensions, with determinant of modulus 1, has a two-to-one homomorphism onto the proper orthochronous Lorentz group $L_{+}^{\uparrow }$ in four dimensions. The space on which $SL(2,\mathbb {C})$ acts is called *spinor space*, and its elements are (one-index) *spinors* with components $\varphi ^A$. Spinor indices like *A* range over 1 and 2, or, commonly, 0 and 1.

The groups $SL(2,\mathbb {C})$ and $L_{+}^{\uparrow }$ respectively preserve determinants of $2\times 2$ complex matrices and the Minkowski metric. Hence we expect that the determinant-forming 2-form in spin space, with components

21 $$\begin{aligned} \varepsilon _{AB}=\left( \begin{array}{c@{\quad }c} 0 &{} 1 \\ -\,1 &{} 0 \end{array} \right) =\varepsilon ^{AB}, \end{aligned}$$

will play the role of the metric. Spinor indices are raised and lowered according to the rule

22 $$\begin{aligned} \varphi ^A=\varepsilon ^{AB}\varphi _B\quad \Leftrightarrow \quad \varphi _A=\varphi ^B\varepsilon _{BA}. \end{aligned}$$

Note that $\varphi _A\varepsilon ^{AB}\ne \varepsilon ^{BA}\varphi _A$. The scalar product of two spinors (with components $\varphi ^A$ and $\psi ^A$) is then defined by

23 $$\begin{aligned} \varepsilon _{AB}\varphi ^A\psi ^B=\varphi _A\psi ^A=-\varphi ^A\psi _A. \end{aligned}$$

If $\varphi ^B$ transforms under $S^A{}_B\in SL(2,\mathbb {C})$, the complex conjugate spinor $\overline{\varphi }^{\dot{B}}$ must, for consistency, transform under the complex conjugate $\overline{S}^{\dot{A}}{}_{\dot{B}}$, and similarly $\varphi _A$ transforms under the inverse of $S^A{}_B$. Dotted (or dashed) indices are used to indicate that the complex conjugate transformations are to be applied. The order of dotted and undotted indices is irrelevant. One can obviously build multi-index spinors, in just the same way that tensors are developed from vectors.

There is considerable flexibility in the relation between vectors and their corresponding two-index spinors $v^{A\dot{B}}$. However in practice it is common to take (with the signature $(-\, -\, -\, +)$) the correspondence

24 $$\begin{aligned} v^1 {\varvec{E}}_1+v^2{\varvec{E}}_2 +v^3{\varvec{E}}_3+v^4 {\varvec{E}}_4 \Leftrightarrow v^{A\dot{B}}= \left( \begin{array}{rr} v^4+v^3 &{} v^1+{\mathrm{i}}v^2 \\ v^1-{\mathrm{i}}v^2 &{} v^4-v^3 \end{array} \right) \end{aligned}$$

between a vector in an orthonormal frame and a spinor in a standard dyad basis (a pair of basis spinors ($o^A$, $\iota ^A$) such that $o_A\iota ^A=1$).

The advantages of the NP spinor formalism are (i) a the spinor equivalent of a real null vector $n^a$ necessarily can be written as $\pm \zeta _{A}\overline{\zeta }_{\dot{W}}$ for some spinor $\zeta _A$, which is very useful in treating physical problems such as gravitational radiation where null (lightlike) vectors naturally arise, and which simplifies some relations involving null vectors and bivectors, and (ii) the somewhat complicated symmetries of the Riemann and other tensors become very simple because multi-index spinors can be expressed as products of completely symmetric spinors and $\varepsilon ^{AB}$ and its complex conjugate.

For example the spinor equivalent of the curvature tensor $R_{abcd}$ can be written as

25 $$\begin{aligned} \chi _{ABCD}\varepsilon _{\dot{W}\dot{X}} \varepsilon _{\dot{Y}\dot{Z}} +\varepsilon _{AB}\varepsilon _{CD} \overline{\chi }_{\dot{W}\dot{X}\dot{Y}\dot{Z}} + \varPhi _{AB\dot{Y}\dot{Z}}\varepsilon _{CD}\varepsilon _{\dot{W}\dot{X}}+ \varepsilon _{AB}\varepsilon _{\dot{Y}\dot{Z}} \overline{\varPhi }_{\dot{W}\dot{X}CD}, \end{aligned}$$

where

26 $$\begin{aligned} \varPhi _{AB\dot{C}\dot{D}}=\varPhi _{(AB)(\dot{C}\dot{D})}=\overline{ \varPhi _{CD\dot{A}\dot{B}}}=\overline{\varPhi }_{AB\dot{C}\dot{D}}, \end{aligned}$$

and

27 $$\begin{aligned} \chi {}_{ABCD}=\varPsi _{ABCD}+{\frac{1}{12}}R(\varepsilon _{AC}\varepsilon _{BD}+\varepsilon _{AD}\varepsilon _{BC}),~~ \varPsi _{ABCD} = \varPsi _{(ABCD)}. \end{aligned}$$

Here *R* is the usual Ricci scalar, $\varPhi$ is equivalent to the traceless Ricci tensor, and $\varPsi$ gives the Weyl tensor. The symmetry of $\varPsi$ enables the notation $\varPsi _J$ for its components, where $J=0\ldots 4$ counts the number of $\iota ^C$ it is contracted with. Similarly one can write the components of $\varPhi$ as $\varPhi _{J\dot{K}}$, where *J* and *K* run from 0 to 2 and count the number of $\iota ^C$ or its complex conjugate in the contractions.

Those coordinate, orthonormal tetrad, and NP formalisms, vector and spinor, are quite commonly used. Tetrads with variable scalar products are less common but sometimes useful (Holmes et al. 1990). The NP formalism has been extended to the GHP formalism (Geroch et al. 1973) and its generalization (Held 1974, 1975), and to a formalism based on a single null vector (Machado Ramos and Vickers 1996) though these have been less often used. (In the case of GHP this is probably because it is best adapted to Petrov type D solutions, and harder to use otherwise.)

That is a far from exhaustive list of computation techniques for components in general relativity, let alone gravity research in other theories. For example, it omits the bivector method (Cahen et al. 1967) closely related to the NP tetrad technique. But it covers most of the component calculations available in packages for CA in GR, and the ideas readily extend, *mutatis mutandis*, to higher-dimensional theories, additional tensorial or spinorial fields, non-commuting objects, geometries with torsion and non-metricity, and so on—examples of which will be mentioned in Sect. 8. In particular, most of the systems which have been or can be used in quantum GR provide facilities for anticommuting or noncommuting objects.

Turning to indicial calculations, suppose we have an expression which is a sum of products of indexed objects (which need not be tensors, though in practice they usually are), each term having the same free indices (since otherwise the expression is not well-formed). We can think of the terms as monomials in the indexed objects. There are two main issues.

The first is to deal with re-ordering or renaming of indices on each monomial: given a monomial one wishes to pick a canonical representative of the class of expressions equivalent to it under the relevant permutations of indices. After transforming each given monomial to canonical form, like terms can readily be collected. In hand calculations the numbers of terms, and of indices on them, are small enough to allow the researcher to manage the issue quite readily, but this will not be true when, for example, looking for relations between scalar polynomial invariants of high order in the Riemann tensor.

The second issue is to deal with simplifications of the sum of terms other than simple collection of like terms: in particular, sum-substitutions may allow combination of monomials or other simplifications. Generalizing the naming by Balfagón and Jaén (1999) and Li et al. (2017) one can call the two issues the monoterm and multiterm problems. [‘Generalizing’ because for example Li et al. 2017 consider only products of Riemann tensors, and the identity (1)].

Not all indicial tensor packages state their methods in detail, so the following is an incomplete account.

The earliest indicial package to be released, ITMS (Bogen and Pavelle 1977), which developed into Maxima’s itensor, used a method of renaming dummy indices, essentially a “systematic labelling scheme” (Hartley 1996), to find the canonical monoterm representatives. Similar methods have been used in later programs, using lexicographic or numerical ordering schemes in the relabelling. For example (Portugal 1999), dummy index renaming can be implemented by first assigning numbers in lexicographic order to indices, and dividing indices into two classes depending on whether a symmetry can move both or one of the dummy pair (see below, McLenaghan’s contribution in MacCallum et al. (1994), and Pollney et al. (1996)), each handled by numerical substitution and reordering: Portugal (1999) noted that Balfagón and Jaén (1998, 1999) used a similar method.

ITMS did not always succeed in intended simplifications (Pavelle 1979). Hartley (1996) stated that none of the packages he had available at the time he wrote his review could successfully deal with all the examples he gave.

Product monomials can be considered as a single tensor via the ‘full reduction method’ of Portugal (1999). For this purpose it is usually assumed that the factors commute, which would not necessarily be true if the objects were indexed differential operators: in fact I do not know of an implementation of the generalization to the non-commuting case. The monoterm problem then has two main descriptions in the literature, either as a group theoretic problem, double coset enumeration, or as a graph theoretic problem. [One may also use rule-based methods (Parker and Christensen 1994), Young tableaux (Peeters 2007a), and so on.]

The double coset formulation, first identified for GR use by Rodionov and Taranov (1989), given a generally used notation by Ilyin and Kryukov , and explained in detail in Martín-García (2008), is as follows. We distinguish two groups that act on the indices: one is the group *S* of symmetries interchanging positions of indices, and the other, *D*, is the group comprising the renaming of dummy indices, raising and lowering by the metric, and exchanging repeated indices. Elements of *S* carry an associated sign depending on whether the exchange is symmetric or antisymmetric.

For a given configuration of indices *g*, the equivalent expressions have index configurations *sgd* where $s\in S$, $d \in D$. The set of these is, in group-theoretic terms, a double coset. In each coset a canonical representative is chosen (perhaps randomly, see Martín-García et al. 2007). Having identified the relevant coset for a monomial, it is then transformed to its canonical representative. There are two well-known permutation group methods for dealing with this problem. One is the algorithm introduced by Butler as developed by McKay (1977) and modified and expounded in Butler and Lam (1985). I understand that the other, the method of Leon (1991), is in principle superior asymptotically for large numbers of indices, but the cross-over is probably outside the range of practical interest for GR. Packages for indicial calculations on tensors typically cite Butler and Lam .

Martín-García (2008) referred to the method he used as the “Butler–Portugal” algorithm, citing Manssur et al. (2002) which combines results from Portugal and Svaiter (2001) and Manssur and Portugal (2001), and giving comparative timings for various implementations. His name for the method reflects the substantial already-cited contributions of Portugal to its development and implementation (cf. also Portugal 1998 where the Gröbner basis technique is proposed for the multiterm work). This method has itself recently been speeded up by improved label renaming and better strategies for the case where subsets of indices are totally (anti-)symmetric (Niehoff 2017 {Mathematica}), using an approach via Penrose’s graphical notation (Penrose 1971, see also the Appendix to Penrose and Rindler 1984: a similar idea was used in Yamashita 1984).

The alternative to the group theory description is as a problem in isomorphism of graphs (Butler and Lam 1985). This description (whose different formulations may use directed or undirected graphs and a variety of graph labellings) is mentioned in Hörnfeldt (1990) and was used in Lim and Carminati (2007) and Li et al. (2017) and references therein. Li et al. (2017) analyse several of the earlier methods in the literature and introduce a new graph-theoretic method.

The multiterm problem concerns relations between components, such as (1). Li et al. (2017) note that although “there are fast monoterm canonicalization algorithms”, “efficient multiterm canonicalization algorithms are still missing”. The objective of the various methods is to express the multiterm expression as a canonically-chosen equivalent expression in terms of canonical monoterms.

Ilyin et al. (1991) and Ilyin and Kryukov (1991, 1996) {Reduce} give an algorithm for the case of linear relations between tensors. They use linear algebra in a Euclidean space of dimension *k*! where *k* is the number of indices involved (the group algebra of the permutation group on the indices). The basis vectors of that space are the set given by writing the relevant tensors with all possible orders of indices. The algorithm then maps any expression to its components in the orthogonal complement of the space spanned by the relations implied by the tensors’ symmetries and the identities arising from dummy index permutations. It uses a ‘triangle’ method of changes of vectors starting with the left sides of identities implied by the symmetries, exchanges of dummies and linear identities.

Portugal’s method (1999), like his associated method for finding a canonical representative of a monoterm, substitutes indices with numerical values and applies an ordering on the numerical lists. Portugal illustrates his method by showing that the algorithm makes the following change

28 $$\begin{aligned} R^{abcd}{{{R^e}_a}^f}_cR_{bfde} \rightarrow R^{abcd}{{{R^e}_a}^f}_cR_{bedf} - \frac{1}{4} R^{abcd}{R^{ef}}_{ab}R_{cedf}. \end{aligned}$$

This example also illustrates the sum-substitution problem as stated above. If one wants the shortest equivalent expression to be the canonical representation, one might prefer to leave the left side of (28) unaltered. However, the same preference would want the change

29 $$\begin{aligned} R^{abcd}{{{R^e}_a}^f}_cR_{bfde} + \frac{1}{4} R^{abcd}{R^{ef}}_{ab}R_{cedf} \rightarrow R^{abcd}{{{R^e}_a}^f}_cR_{bedf} \end{aligned}$$

to be made. Similar comments apply to the example given in Kavian et al. (1997).

I have not yet seen a description of an algorithm for non-linear identities such as that arising from the Ricci identity for the curvature:

$$\begin{aligned} {{R_{cde}}^f}_{;[ab]} = {R_{cab}}^k{R_{kde}}^f + {R_{dab}}^k {R_{cke}}^f + {R_{eab}}^k {R_{cdk}}^f - {R_{kab}}^f {R_{cde}}^k, \end{aligned}$$

other than the approach of Invar (see Sect. 8.1.1 and [C38]), which expresses multiterm expressions in terms of a minimal basis of monoterms, and the rule-based methods of Christensen (1998) {MathTensor}.

As mentioned earlier, the procedures used in Stensor (see above and Sect. 7.4) seem rather effective but are incompletely documented. Kavian et al. (1997) {Mapletensor} used genetic algorithms, a non-deterministic and stochastic method. Further methods for similar and related problems are discussed in Harris (1999), Ilyin et al. (2000) using Java, and Liu (2017).

## Requirements of CA for GR

Programs for gravity need some standard CA features, like

- Good simplification routines
- Good control of substitutions
- Differentiation
- A reasonable range of known mathematical special functions, and facilities to add others.
  Depending on their intended uses, they may not need:
- Polynomial factorization
- Integration and solvers for differential equations
- Numerical and graphical features.

CA for GR clearly requires tensor algebra and calculus. More precisely it needs facilities for handling indexed geometric objects, not all of which are tensors (though for brevity I shall refer to them all as tensors in the subsequent discussion). The more fully-featured systems offer user-friendly facilities for extending both the set of objects and the operations on them. For GR, one may need to go beyond general facilities for tensor algebra, both in component and in indicial calculations, as I now discuss (see also Korolkova et al. 2013). It is also useful to be able to handle differential and algebraic identities like the Ricci identity, requiring “sum-substitution”, which, as discussed above, pose hard problems for CA systems.

The exact choice of facilities offered depends on the purposes the designer had in mind: examples of facilities available in some packages and not others include classification of the Weyl (conformal curvature) tensor in general relativity into Petrov types, and functional differentiation of Lagrangians. Algorithm choice can markedly affect efficiency (see below, McLenaghan’s contribution in MacCallum et al. 1994; Pollney et al. 1996).

For component calculations, efficient methods for storage and retrieval of components that take into account the symmetries on indices are desirable. For example, in a four-dimensional spacetime the curvature tensor $R_{ijkl}$ has 256 components but all except (at most) 20 of these can be expressed linearly in terms of the others: this is one reason why, rather than using arrays or matrices, many systems employ a functional programming paradigm to define tensors as functions of their indices, the function then returning the value of the component for the given index values. To make such definitions of tensors efficient one needs combinatorial algorithms to deal with the symmetries. The more extendible packages provide ways to specify the symmetries of new objects that will automatically generate good storage and retrieval methods.

Input formats still have elements of the FORTRAN style of early systems, mainly because many users find it quicker and easier to type input data as ASCII characters along a line than to move up and down between lines to compose a formula or to search in a palette for the symbol one wants. One specific issue is how to indicate index positioning in one’s input. Mapletensor (Kavian et al. 1996) used upper and lower case letters while MathTensor (Parker and Christensen 1994) used “ua” and “la”. Some systems have used  -like notations such as $\wedge$a and _a or, (e.g.) as in the itensor package in Maxima, used $-a$ for contravariant and *a* for covariant indices. Classi (see Sect. 7.4) has a rather flexible but somewhat verbose way to define index positions when a tensor is defined, and treats different index positions for the same object as defining different tensors (not unreasonable since the component values will in general be different). This, and other issues concerning the relation of input notation and textbook notation, may be important factors in users’ choices of package.

The specific objects and functions required by a user are of course problem-dependent. For component calculations they may include Petrov and Segre classification, coordinate and tetrad transformations, the ability to handle alternative sign conventions, the computation of sets of scalar or Cartan invariants (as defined in MacCallum 2015), covariant differentiation to any order, the handling of Lie algebras, geodesic equations, auxiliary fields (Maxwell, Yang–Mills and so on), a wide range of geometric object types, operations such as Hodge duality, functional differentiation, perturbation expansions, complex numbers and functions, and flexibility in metric, connection and so on (perhaps allowing multiple metrics to be worked on simultaneously). No single system at present (as far as I know) offers all possible such facilities for geometry and gravity, though some come close.

It is also important to choose algorithms with care. Sometimes this takes one in a different direction from general research in CA. That research tends to be devoted to finding algorithms good for asymptotically large dimension *n*, while CA in GR usually requires efficient algorithms for small dimension *n*.

As a simple example in algorithm choice, the direct use of the textbook formulae (9) and (10) for calculating the curvature from the metric in coordinates is inefficient, compared with $[jk,\ell ] := g_{j\ell ,k}+g_{k\ell ,j}-g_{jk,\ell }$ and

30 $$\begin{aligned} R_{ijkl} = {\frac{1}{2}}[g_{i\ell ,jk}+g_{jk,i\ell }-g_{ik,j\ell }- g_{j\ell ,ik}] +g^{mn}\{[jk,m][i\ell ,n]-[j\ell ,m][ik,m]\}, \end{aligned}$$

Hartley (1996). There are two main reasons. The formula (30) avoids differentiating the inverse metric $g^{ij}$, which is the ratio of one polynomial of degree 3 in the components of $g_{jk}$ and another of degree 4, and $R_{ijkl}$ has more symmetry than ${R^i}_{jkl}$ so fewer components need to be stored. For this sort of reason the intermediate quantities a system calculates may not be the ones users expect on the basis of textbook formulae, but instead may follow a path chosen for efficiency reasons. (For an example where inefficient intermediate steps seem to have been used, see MacCallum 1989.)

A second example is provided by the discussion in Allen et al. (1994). A textbook computation of Newman–Penrose quantities might work by

31 $$\begin{aligned} {C^a}_{bc}= & {} ({h^a}_{i,j}-{h^a}_{j,i}){h_b}^i{h_c}^j, \end{aligned}$$

32 $$\begin{aligned} \varGamma _{abc}= & {} {\frac{1}{2}}(C_{cab}-C_{abc}-C_{bca}), \end{aligned}$$

where ${h^a}_i$ are the components of the basis vectors. Using Jacobi’s theory of minors to re-express the inversions of *h*, Allen et al. obtained

33 $$\begin{aligned} {C^a}_{bc} = \left( {h^a}_{i,j} \delta ^{ijkp}_{bcde}{h^d}_k{h^e}_p\right) \Big /\det \left( {h^f}_q\right) , \end{aligned}$$

leading to a speed up by a factor 2 in four dimensions. They then found that even better was to do a once-for-all evaluation of (33) and write (for example) the NP coefficient $\mu$ as

34 $$\begin{aligned} \mu = {\frac{1}{2}}e^{ijkp}[n_{i,j}\ell _kn_p +m_{i,j}\bar{m}_kn_p - \bar{m}_{i,j}m_kn_p]/(i\sqrt{-g}) \end{aligned}$$

leading to another speed up of factors 2 to 5 and a saving in memory of 65% or more. The method using (33) is an example of the Cartan package for Maple which was designed to handle a general tetrad, while the one using (34) was part of the Debever package modelled on Debever’s null frame formalism.

The efficiency of algorithms is important, but a user who does not have heavy and/or repeated calculations to make may choose to meet the need for the user to understand the mathematics by preferring one of the less sophisticated programs listed below, which may thus still be of value. In particular, such a user might prefer one of the packages which more or less directly implements textbook formulae. To achieve input notation mirroring standard formulae, one usually has to define operators as “infix” rather than “prefix” and use textbook tokens for their names, so that one can write (e.g.) a$\wedge$b rather than wedge(a, b).

For indicial tensor calculus, the most critical aspect is the handling of dummy indices and symmetries, discussed in Sect. 4. Other features desirable in an indicial package include the ability to deal with subspace splitting and with additional fields (requiring simultaneous use of two or more index sets); functional differentiation with respect to indexed objects; substitutions for indexed objects, for example substituting from (9) into (10) to express the curvature directly in terms of the metric; ‘tensor compiling’ (the generation of code for component computations from the indicial formulae); and the handling of commutation rules for non-commuting objects. It is worth noting that although indicial tensor calculus does not give rise to the issues concerning efficient storage and retrieval of values by component calculators which were discussed above, the combinatorial algorithms required to do that are similar to those needed in transforming a monoterm to canonical form.

## GR packages for general purpose systems

Here I shall briefly outline the available packages for GR and/or differential geometry within each of the main general purpose CA languages (Macsyma/Maxima, Maple, Mathematica, Reduce). To the best of my knowledge there is no GR package for Axiom or MuPAD, or for Sage other than SageManifolds (see Sect. 3.2.5) and those for the Maxima within Sage. There was a package, muTENSOR, for muMATH, which was closely related to the Reduce package REDTEN (Dyer and Harper 1988). I do not know of packages that worked with Derive.

Some of the packages for general purpose CA systems are automatically included in the standard distributions of those systems. The other packages mentioned here are almost all freely available over the internet, even those written for the commercial general purpose CA systems. I believe the exceptions are atlas 2 (for Maple and Mathematica$^{\textregistered }$^11^) and the Mathematica$^{\textregistered }$ programs MathTensor and Tensorial. Some others have at various times charged a fee or been shareware (i.e. relied on optional donations) but I have not recorded those histories.

A few packages seem to me to have particularly interesting features or be of special importance because of their widespread use or the facilities they offer (or both) and I have given these separate subsubsections of their own. The selection of these is of course entirely subjective.

### Macsyma/Maxima

#### Current Maxima packages

Maxima ([C2], [C7]) comes with four relevant packages. Of these, ctensor and itensor were inherited from DoE-Macsyma, but “in various stages of disrepair” (Toth 2005). A new module atensor was added to achieve parity with the commercial version of Macsyma (Toth 2005). The Maxima manual provides descriptions of the four packages. They are:

- **atensor** For algebraic tensor manipulation in the sense of objects in, for example, a Clifford, Grassmannian or symplectic algebra. “The essence of atensor is a set of simplification rules for the noncommutative (dot) product operator (“.”).”
- **cartan** This, which is described under “Functions for differentiation” in the manual, implements the exterior product and Lie and exterior differentiation for differential forms. It was originally written by Frank B. Estabrook and Hugo D. Wahlquist.
- **ctensor** For component calculations in coordinates or frames. It was extensively rewritten in 2004. It can Taylor expand tensors, compute Petrov type (by the method of Pollney et al. 2000c, which adapts that of Åman et al. 1991), and include torsion and nonmetricity.
- **itensor** For abstract (indicial) tensor manipulation. In itensor a tensor is represented as an “indexed object”, a function of 3 groups of indices which define the covariant, contravariant and derivative indices.

#### Earlier Macsyma/Maxima packages

**GEOCALC** (Moussiaux and Tombal 1987; Tombal and Moussiaux 1989) was based on the Geometric Calculus of Hestenes (1986), which used Clifford algebra based on a “fiducial” orthonormal frame. GEOCALC could use those methods to calculate the curvature from a metric.

Early versions of Stensor (see Sect. 7.4) were also implemented in Macsyma, but I do not know of an available source for that code.

### Maple

Maple has during its evolution had a succession of GR or differential geometric packages distributed either as part of the system or with the system in its share library, or made available independently. At one time there seems to have been more than one “tensor” package distributed with the system. The most recent version I have (Maple 2017) includes a physics package which is said to be better integrated with other Maple facilities, such as the DifferentialGeometry package, than past packages: it was presented in a “webinar” [C17] and the worksheet shown in that webinar is also available [C18]. The physics package is an in-house product, with work led, I believe, by Edgardo Cheb-Terrab.

#### Current Maple packages

To the best of my knowledge these are the other current Maple packages: note that GRTensorII (see below) could still be included in this list although it has been superseded by GRTensorIII.

- **Atlas 2:** This is a commercial product for Mathematica$^{\textregistered }$ and Maple. (See the footnote in Sect. 5 about availability as of February 2018. As far as I know this package is not related to the ATLAS numerical linear algebra software project.) The website [C19] said it was updated to Maple 2015. The overview at the website gave information on the capabilities and appearance. The summary there is “Atlas 2 for Maple is a powerful Maple toolbox for performing calculations in the general area of differential geometry: from formulating and solving 2D/3D problems to working with an N-dimensional manifold as a whole. Atlas 2 allows you to concentrate on differential geometry problems, but not on the programming. Atlas 2 uses standard differential geometry notations which allow you to always get output as you expected.”
- **Canon:** (Manssur and Portugal 2004) [C71], program ADSP. This package provides indicial tensor methods especially for the monoterm problem. It was used to support the Invar work (see Sects. 6.3.4 and 8.1.1). It is also used by TensorPack (see below), whose distribution pack includes a 2008 version of Canon.
- **DifferentialGeometry:** [C20] is a package developed by Ian Anderson and Charles Torre: as described in Anderson and Torre (2012) it is a component-oriented package. It performs fundamental operations of calculus on manifolds, differential geometry, tensor calculus, General Relativity, Lie algebras, Lie groups, transformation groups, jet spaces, and the variational calculus. There are self-explanatory subpackages for: GroupActions, JetCalculus, and LieAlgebras. The “Tensor” subpackage provides support for advanced GR applications. The “Tools” subpackage is for developing new applications. The breadth of coverage has led to the use of DifferentialGeometry in a considerable number of papers other than the examples in GR, notably in relation to differential equations and systems. The latest versions are being developed independently, but an earlier version is included with Maple, and is used by the physics package.
- **Exterior:** Written by Mark Hickman, this package’s initial purpose was to investigate Lie symmetries [C21] but it has now been extended to cover various (other) GR calculations. “For example, the computation of the curvature of the Schwarschild metric in a moving frame, coordinate frame and a null tetrad”.
- **Finsler:** This package for calculations in Finsler geometries was developed from a package in Riemann (see below) and written by Rutz and Portugal (2001, 2003). It provided coordinate component calculations and some indicial facilities. It has been modified by Youssef and Elgendi (2014) so that it works not only with “the geometric objects associated with Cartan connection but also those associated with Berwald, Chern and Hashiguchi connections in any dimension”: [C71], program AERE.
- **GRTensorIII:** [C22], which was released in early 2017, updated and upgraded GRtensorII (see below). It provides component calculations, NP calculations, and calculations for hypersurfaces and junction conditions. The authors of this and its predecessor are Kayll Lake, Peter Musgrave and Denis Pollney. It can now export a metric formatted for DifferentialGeometry (see above).
- **Riemann:** (Portugal and Sautú 1997) [C71], program ADGP. “The package has been developed for tensor component calculations in General Relativity and for some tensor abstract manipulations. It allows the user to perform tensor algebra operations, such as addition, multiplication and contraction of tensors. It is also possible to create new tensors with defined symmetries and to apply Maple functions to the tensor components.” It was based on Tensorcalc (Portugal 1997), which had similar indicial and component capabilities, and originally ran in Maple V.3. Riemann is used by, and can be downloaded with, TensorPack, and by Finsler.
- **TensorPack:** (Huf and Carminati 2015) [C23] is an indicial tensor calculation package (the authors call this the covariant index format). “TensorPack is based on the Riemann and Canon tensor software packages and uses their functions to express tensors in an indexed covariant format. TensorPack uses a string representation as input and provides functions for output in index form. It extends the functionality to basic algebra of tensors, substitution, covariant differentiation, contraction, raising/lowering indices, symmetry functions and other accessory functions.”

#### Earlier Maple packages

Except for GRTensorII, the following earlier packages are as far as I know not available, or not usable, or not maintained, for the current version of Maple:

- **bianchi:** This module was part of the Maple V library and enabled classification of three-dimensional Lie algebras into Bianchi types, important in exact solutions especially for cosmology.
- **Cartan:** This package provided general tetrad calculations (McLenaghan in MacCallum et al. 1994; Allen et al. 1994). It was in the Maple V library.
- **Debever:** This provided calculations using Debever’s null frame formalism (McLenaghan in MacCallum et al. 1994; Allen et al. 1994). It was in the Maple V library.
- **difforms:** A differential forms package present from Maple V (Char et al. 1991) until at least Maple 9.5.
- **fjeforms:** A differential forms package by F. J. Ernst that was present in Maple V.
- **forms:** This provided differential form calculations (Lang 1993). It was written to overcome the limitations of the difforms package for studies in the equivalence problem and related areas.
- **GHP:** A package implementing the GHP (Geroch–Held–Penrose) formalism (Carminati and Vu 2001; Vu and Carminati 2003).
- **GRtensorII:** This [C24] was a rather widely used package, developed from the earlier GRtensor (described in d’Inverno 1980) and now superseded by GRTensorIII. It is “a computer algebra package for performing calculations in the general area of differential geometry. Its purpose is the calculation of tensor components on curved spacetimes specified in terms of a metric or set of basis vectors. The package contains a library of standard definitions of a large number of commonly used curvature tensors, as well as the Newman–Penrose formalism. The standard object libraries are easily expandable by a facility for defining new tensors. Calculations can be carried out in spaces of arbitrary dimension, and in multiple spacetimes simultaneously.” Additionally to material available at [C24], Pollney et al. (2000b) provides a useful introduction.
  Although essentially not updated since Maple 11, GRTensorII is still available and its website gives instructions on running it under recent Maple versions. It supported a package for junction conditions, GRJunction (Musgrave and Lake 1996, 1997), and was used in construction of an online database of exact solutions [C74], which had access provided by a graphical interface GRtensorJ (Ishak et al. 1999).
- **LUCY** (Schray et al. 1996) was “a MAPLE program that exploits the general theory of Clifford algebras to effect calculations involving real or complex spinor algebra and spinor calculus on manifolds in any dimension.”
- **Mapletensor:** This was a system for both indicial and component calculation (Kavian et al. 1996, 1997). I understand that the lead author, Kavian, tragically died before the planned developments in the cited papers were complete.
- **NP** and the related program **NPspinor** to convert spin equations to dyad form. These packages (Czapor and McLenaghan 1987; Czapor et al. 1992, and McLenaghan’s contribution in MacCallum et al. 1994) provided the Newman–Penrose tetrad and spinor formalisms.
- **NPtools:** This package had three parts: (i) conversion between orthonormal and null tetrads; (ii) an implementation of the full Lorentz group action; and (iii) the Petrov–Plebanski method of classifying the Ricci tensor (Cyganowski and Carminati 1998). Available in the CPC program library [C71] as ADJM.
- **ORTHOFRAME/oframe:** This package provided orthonormal tetrad calculations in the Ellis–MacCallum form (see above) (Van den Bergh 1988). It was distributed with Maple V.
- **PROCRUSTES:** is “a package of routines for the computer algebra system Maple which supports the explicit determination of the geometric quantities, field equations, equations of motion, and conserved quantities of General Relativity in the post-Newtonian approximation.” (Puetzfeld 2006), [C71], program ADYH. Tested with Maple 10.
- **Riegeom:** “This paper describes a new package for abstract tensor calculation.
  Riegeom can efficiently simplify generic tensor expressions written in the indicial format. It addresses the problem of the cyclic symmetry and the dimension dependent relations of Riemann tensor polynomials.” (Portugal 2000), [C71], program ADLM.
- **tensor:** This was a package for coordinate component calculations that formed part of MapleV.4 (Chu et al. 1996). A package of this name, presumably the same, was still present in Maple 14. The same package name and some of the command names had earlier been used in a library command (Char et al. 1991).

### Mathematica$^{\textregistered }$

#### Current Mathematica$^{\textregistered }$ packages

Reflecting its commercial success in sales to physics departments, Mathematica$^{\textregistered }$ has the largest number of packages for gravity or differential geometry of any of the main general purpose systems. The following are available:

- **atlas2:** This is a commercial product for Mathematica$^{\textregistered }$ and Maple. (See the footnote in Sect. 5 about availability as of February 2018.) The website [C25] says it is updated to Mathematica 10. The overview at the website gives information on the capabilities and appearance. The summary there is “Atlas includes a full list of functions for calculating common differential geometry problems, deploys results in standard math notations to maximize your productivity, and automates solving path of your tasks allowing to concentrate on ideas. Even if you’re an expert, you solve differential geometry problems faster with Atlas! Atlas 2 for Mathematica includes Atlas Palette that is integrated with DG Library Visualization and Manipulation Atlas offers powerful functionality for visualization of multidimensional differential geometry objects uniquely integrated with Mathematica. Atlas provides access to Differential Geometry Library with hundreds of objects and continuously growing data collection.”
- **ccgrg:** (Woszczyna et al. 2016) [C26]. The “characteristic feature of the ccgrg package is the specific coupling between the functional programming and the Parker-Christensen index convention [i.e. the one introduced in MathTensor, see below]. This causes that no particular tools to rising/lowering tensor indices neither to the tensor contractions are needed. Tensor formulas are written in the form close to that of classical textbooks in GR, with the only difference that the summation symbol appears explicitly. Tensors are functions, not matrixes, and their components are evaluated lazily.” From private discussion, I am aware that the authors of ccgrg made a detailed study of other existing Mathematica$^{\textregistered }$ packages when developing theirs. Woszczyna et al. (2016) also reported an implementation of similar features in Python [C57].
- **diffgeo:** This is one of a set of packages by Matthew Headrick [C27], “a package for doing GR-type tensor algebra and calculus. Compared to other such packages I know, it is easy to use and fairly comprehensive in the number of functions defined.” Other programs on the same web page deal with Virasoro algebras, Grassmannians (this, developed with Jeremy Michelson, seems to be the same as the grassmannOps package listed at [C38]), approximations to Calabi–Yau manifolds and so on.
- **EDC/super EDC:** “Exterior Differential Calculus” [C28] is a differential forms package offering quite extensive facilities.^12^ An earlier version is available in the Wolfram Library Archive. It has been used in calculations of Lie algebras and cohomology. The extension superEDC can handle superalgebras.
- **EFTofPNG:** This is a code for high precision Feynman computation in the Effective Field Theory of Post-Newtonian Gravity (Levi and Steinhoff 2017) [C29]. “The code covers the current state of the art PN accuracy including spinning components in the merging compact binaries. Its final unit computes observables useful for the waveform modelling, and serves as a pipeline chain for the wave templates.”
- **EinS** (Klioner 1998; Grabmeier et al. 2003) [C30] is a “package allowing one to perform operations with indexed objects, which may or may not be tensors. The main application field of EinS is computations with indexed objects involving implicit (Einstein) summations (EinS stands for ‘Einstein Summation handler’). The idea of the package was to create a simple (EinS is a relatively small package consisting of approximately 3000 lines of code), flexible package which would be easy to alter for solving any problem involving indexed objects”. The current version is 2.7.
- **geodesicCOMMENTED:** “A procedure is developed for obtaining the metric tensor explicitly from the Christoffel symbols. The procedure is extended for determining if a second order quadratically semi-linear system can be expressed as a system of geodesic equations...” (Fredericks et al. 2008), [C71], program AEBA.
- **GRtensorM** [C24] is a restricted version for Mathematica of the GRTensorII package for Maple (see above), written by the same authors.
- **GRworkbench** is of rather different character from all the other CA in GR packages. Thus a fuller description appears in Sect. 6.3.3.
- **Kranc** is a module for “turning a tensorial description of a time dependent partial differential equation into a module for the Cactus computational toolkit”, used in numerical general relativity. It can output parallelized C or Fortran code, performing some optimizations (Husa et al. 2006) [C31]. Since initial release it has been “actively developed by Ian Hinder, Erik Schnetter and Barry Wardell.” It forms an integral part of the publically available EinsteinToolkit [C72], and was used to generate its standard BSSN codes.
- **MathGR** [C32] (Wang 2013) is a package to manipulate tensor and GR calculations with either abstract or explicit indices, simplify tensors with permutational symmetries, decompose tensors from abstract indices to partially or completely explicit indices and convert partial derivatives into total derivatives.
- **MathTensor:** This (Parker and Christensen 1994; Christensen 1998) was the earliest Mathematica package for GR that I know of, and was (is?) a commercial product. It has indicial and component capabilities, and a differential forms module. Owing to a fire, as of 2017 it had no website but was available from its co-author, Steve Christensen, at sunfreeware@gmail.com. It is known to have worked in Mathematica$^{\textregistered }$ 10 and is believed to work in the current version.
- **RGTC:** “Riemannian Geometry and Tensor Calculus” [C35] is a component calculation package with the standard facilities. It can classify the curvature in 4 dimensions, and provides calculations in null tetrads and the Newman–Penrose formalism. It can combine with EDC (see above) for calculations in arbitrary frames.
- **Ricci**, [C33], is intended “for doing symbolic tensor computations that arise in differential geometry”. From its website’s description it appears to be a rather fully-featured indicial tensor manipulator, which recommends MathTensor for component calculations. The latest Mathematica version for which its website, updated in 2016, says it has been tested was 5.
- **Ricci** (another one!) written by Juan M. Aguirregabiria [C37] provides coordinate component calculations. It is a partner of Tetrad (see below).
- **TensoriaCalc** [C36] by Yi-zen Chu “tackles (semi-)Riemannian tensor calculus problems encountered in general relativity, cosmology, and field theory. Currently, it calculates geometric objects—Christoffel symbols, the Riemann curvature tensor, Ricci tensor and scalar, etc.—given a metric and the relevant coordinates; and performs basic operations such as covariant derivatives of tensors”. It was updated to Mathematica 10.
- **Tetrad** written, like the second “Ricci” above, by Aguirregabiria [C37] provides tetrad component calculations.
- **xAct:** “Efficient Tensor Computer Algebra for Mathematica” is a collection of packages for fast manipulation of tensor expressions [C38], providing both indicial and component calculations. The basic ideas are said to have been taken from the abstract index approach (see Sect. 4). It has attracted a significant group of contributors and been used in a number of projects. Thus it has developed into a rather fully-featured system and merits a fuller description below (Sect. 6.3.4).

This list should perhaps include FeynCalc [C39], a package for Feynman diagrams, which may be useful in quantum GR. In addition there is an unnamed program dealing straightforwardly with the 3+1 formalism in coordinates (Hasmani and Panchal 2015): this reference cites other programs by the same authors. Dai-Ho Park’s “Indicial Tensor Package Using Notebook Interface” packages [C40] are another set of programs apparently without an overall name; they “contain routines for manipulating indical and component tensors in General Relativity and Kaluza–Klein Theory. References and a few examples are included in notebooks.”

#### Earlier Mathematica packages

- **CARTAN:** This package is described in Soleng (1996), but I have not been able to find a current source. The author left research in GR in 1997. Despite that, the package was extended to, and used in, Weyl–Cartan spacetimes (Babourova et al. 2009).
- **Differential Forms:** An exterior forms package written by F. Zizza [C41]. Last known to have been updated for Mathematica 6.
- **EinsteinTensor:** This package, written by Pekka Janhunen, derived curvature from a metric in matrix form [C43]. Last updated, it appears, in 1992.
- **EVOL and BOLTZ** These provided a 3+1 treatment of the field equations together with the Liouville operator for the relativistic Boltzmann equation (Salgado 1994) [C71], program ACTG. The programs used relativistic transport theory as formulated by Lindquist for neutrino propagation.
- **GREAT:** Like EinsteinTensor (see above), on which it was based, GREAT [C42] calculated curvature from a metric matrix. It was written by Tristan Hubsch: the quoted URL gives a revision date of 2003.
- **grt:** The facilities of grt [C44] were modelled on GRtensor. The author, Pascal Vaudrevange, left academia in 2012.
- **Tensorial** was “a general purpose tensor calculus package for Mathematica” written by Renan Cabrera, David Park, and Jean-François Gouyet, with indicial and component capabilities. I have been unable to download a copy, though there is a webpage [C45], with a latest date 2007, stating it was updated to Mathematica 6.0.
- **Tensors:** This provided tensor facilities based on multilinear algebra and matrix methods (Ruíz-Tolosa and Castillo 2004) but the software download page at [C46] still refers to Mathematica$^{\textregistered }$ 4.0. It appears to have been focused on tensors in Euclidean space.
- **TTC:** “Tools of Tensor Calculus”, written by A. Balfagón, P. Castellví and X. Jaén (Balfagón and Jaén 1998, 1999; Castellvií et al. 1994), provided indicial and component calculations for tensors including handling of submanifolds and changes of coordinates. I have been unable to locate a current website for TTC.

#### GRworkbench

A number of programs have set out to provide graphical or numerical tracing of geodesics in gravity, typically in general relativity, for example those mentioned below. GRworkbench is unusual in providing a CA interface to the numerical part of the work: it is particularly aimed at enabling the investigation of global structures. The idea of doing so by tracing geodesics was at the heart of the surprising and impressive results of Scott and Szekeres (1986a, b). One subsequent use was to investigate a claim that the mass of the Milky Way could be measured using an interferometer on Earth (Moylan 2003).

Some of the other programs which provide ray-tracing in curved space are:

Motion-4D (Müller 2014b), written in C++ but using GRTensorII to create the metric input data in the Metric classes, [C71], program AEEX;

GeodesicViewer (Müller 2011) which gave graphical representations using Motion-4D, [C71], program AEFP;

Gyoto (Vincent et al. 2011) [C76] using the Yorick language and Python.

GpuRay4D (Kuchelmeister et al. 2012) using a GPU, [C71], program AEMV; and

GeoVis, aimed at visualization of objects (Müller 2014a), [C71], program AESY.

GRworkbench has undergone evolution through a set of substantially different designs. A first version used a custom graphical user interface (Evans 2000). This was then replaced by a library in the Lax language, which gave rise to a version in C++ (Searle 1999; Evans et al. 2002; Moylan et al. 2005a, b). There were limitations of this approach and a set of options were considered for a new version (Lewis 2014), the final choice being to implement in Mathematica$^{\textregistered }$. The numerical part of the code uses lazy evaluation to continue the computed geodesics from the point already reached.

#### xAct

xAct is a suite of packages, first released in 2004: the version currently available at [C38] is 1.1.2, released in August 2015. Four packages act as a kernel for the rest. They are:

- xCore: generic programming tools
- xPerm: manipulation of large groups of permutations (Martín-García 2008)
- xTensor: abstract tensor computations, the “flagship of the system”
- xCoba: component tensor computations.

xPerm provides a major component of the indicial manipulation capabilities, the monoterm canonization discussed in Sects. 4 and 8.1.1.

There are some key applications and a number of contributed modules which are supplied with the overall package. For an up-to-date list see [C38]. The main application modules are:

- xPert: high-order perturbation theory in GR (Brizuela et al. 2009)
- Harmonics: tensor spherical harmonics (see Brizuela et al. 2006)
- Invar: provides a list and basis of all scalar polynomial invariants of the Riemann tensor and its covariant derivatives up to the 12th (Martín-García et al. 2007, 2008): see Sect. 8.1.1
- Spinors: spinor computations in GR (García-Parrado Gómez-Lobo and Martín-García 2012)

The Spinors module and the contributed SpinFrames module provide NP and GHP formalisms.

The contributed modules extend the main ones in various directions. One giving a range of extra facilities that have been quite widely used is xTras (Nutma 2014), which was “field-theory inspired”. Capabilities of xPand, which extends xPert, are mentioned in Sect. 8.2.2. Cyril Pitrou pointed out to me that one can combine xPert with the variational derivative operator of xAct to obtain field equations from any perturbed action.

Although I believe the original lead author of xAct, Martín-García, now works for Wolfram Inc (the sort of circumstance that has led to other packages becoming defunct), there are a body of other developers still involved, and the package has attracted a considerable body of users, as shown by the extensive bibliography of articles using the package [C66], so it seems likely to be maintained in future. It certainly seems to be one of the most up-to-date and fully featured packages at present. Its website lists over 300 applications papers: a number of its recent applications are mentioned below.

### Reduce

#### Current Reduce packages

The standard Reduce distribution provides 5 compiled packages which can be or have been used in GR (and a couple more are among the contributed packages circulated with Reduce). Details can be found at [C6]. In brief the 5 are:

- **atensor:** An indicial tensor package (Ilyin and Kryukov 1994, 1996): see Sect. 4.
- **dummy:** This provides reduction of sets of indices (such as the indices in an indicial tensor expression) to canonical form (Dresse 1993a, b; Dresse and Henneaux 1994). It is or was used in the CANTENS package by Hubert Caprasse (see below).
- **eds:** (Hartley and Tucker 1991; Hartley 1997). This “provides a number of tools for setting up and manipulating exterior differential systems and implements many features of the theory. Its main strengths are the ability to use anholonomic or moving frames and the care taken with nonlinear problems.”
- **excalc:** This provides a range of facilities for calculations written in terms of differential forms. Because it has been used in a number of applications described later, a slightly fuller description is given in Sect. 6.4.3.
- **susy2:** “This package deals with supersymmetric functions and with the algebra of supersymmetric operators in the extended $\hbox {N}=2$ as well as in the nonextended $\hbox {N}=1$ supersymmetry.”

In addition there are a number of packages for calculations in GR which are available but are not part of the main Reduce distribution:

- **Dimsym**: Written by James Sherring, Geoff Prince and Michael Jerie, this is primarily for the determination of symmetries of differential equations [C47]. It has been used for such problems in GR (Jerie et al. 1998) and has an interface to excalc (see above)
- **GRG3.2:** This package (Zhytnikov 1994) [C50] and **GRG**$_\mathbf{EC}$ (Tertichniy and Obukhova 1997) evolved from GRG 3.1 (Obukhova et al. 1992). It is a component calculator providing a wide range of facilities, including spinors, Riemann–Cartan spaces, differential forms, and indicial operations. One unusual feature is that it can output results in the formats of several other CA systems.
- **REDTEN:** This package (Dyer and Harper 1988, [C49]) was updated to Reduce 3.8: it builds correctly in the current CSL version of Reduce. Redten has indicial and component features and provides NP and frame packages.
- **RicciR:** (formerly TMP—Tensor Manipulation Package) is an indicial tensor package (Kadlecsik 1992, 1996). To obtain the code, see [C48]. It has not been updated since about 1997.
- **TAVI:** (Demichev and Rodionov 1985) provided an extension from the spacetime geometry to that of a higher-dimensional fibre bundle of Yang–Mills type, enabling calculation of the geometry of the group quotient space *G* / *H*. It is still available at [C71] as program AADJ.

The Reduce distribution set now also includes Ortocartan (see Sect. 7.3) in the “contrib” directory.

It should be noted that because one can build combined Reduce/Sheep systems, Sheep (Sect. 7.4) could nowadays be considered a Reduce package. The Reduce/Sheep combined versions offer a non-commercial system competitive in power and features with the larger Maple or Mathematica$^{\textregistered }$ packages.

#### Earlier packages for Reduce

The earlier Reduce packages I know of are:

- **CANTENS:** This is available in the packages/assist subdirectory of the main Reduce distribution [C6], but it appears not to have been maintained recently. Written by Hubert Caprasse, it uses the assist and dummy packages, provides indicial and component calculations, and can handle more than one space simultaneously.
- **GENRE:** This was an overall name for a suite of programs in Reduce 2 which supplied coordinate and NP component calculations, perturbation methods, and some unusual facilities like null hypersurface geometry (Dautcourt et al. 1981; Dautcourt and Jann 1983).
- **GRLIB:** This suite of programs for component, tetrad and NP calculations was written by the late Dermott McCrea and is described in MacCallum et al. (1994). The programs are still available [C51] but somewhat outdated (for example, date from before Reduce changed default case). While later programs offer more, these programs are easy to understand and adapt, and both the code and the description have been found useful in later research (Friedrich Hehl, private communication).
- **RTENSOR** was “a package for handling algebraic expressions having dummy indices and symmetries with respect to index permutations” (Rodionov and Taranov 1988a, b, 1991).

In addition an unnamed program for Newman–Penrose calculations is listed in full in Esteban and Ramos (1990).

#### EXCALC

Input and output for the differential forms in this package are close to textbook format and it is therefore very easy to use. It can deal with indexed objects and has many geometric objects and ideas built in. As examples, Table 1 shows some of the near-textbook notations.

**Table 1:** Some EXCALC notation

Input notation	Textbook notation	Meaning
	$\wedge$	Exterior multiplication
_|	$\lrcorner$	Contraction of a vector with a form
|_	$$\mathcal{L}$$	Lie derivative
@	$\partial$	Partial derivative
#	*	Hodge dual
d	d	Exterior derivative
d t	dt	One-form, exterior differential of *t*

The package knows the duality of natural basis elements such as *dx* and @(*x*). A coframe and metric can be defined together by (e.g.)

coframe e r=d r, e(ph)=r*d ph with metric g=e(r)*e(r)+e(ph)*e(ph); and so on.

The differential forms can themselves bear indices, as in Eqs. (13) and (14). Given a Lagrangian *n*-form, EXCALC can compute variational derivatives, including specification of boundary conditions, and calculate conserved quantities induced by symmetry operators. Coordinate or tetrad calculations are defined via specification of basis one-forms as in the example above.

## Standalone software for CA in GR

As well as packages built on general purpose CA systems there are special purpose systems. Some were of limited availability or applicability (for example, there was a quite impressive one, TINMAN, written as part of a Master’s thesis I examined in 1996, which was designed to run on the original 640K DOS PCs). There have also been announcements of potentially useful packages, e.g. Dreitlein and Sauer (1990), which I have not (yet) found mentioned in later applications. I omit those. The standalone CA for GR packages which I believe current are:

### Cadabra

From the website [C56]:

“Cadabra is a symbolic computer algebra system (CAS) designed specifically for the solution of problems encountered in field theory. It has extensive functionality for tensor computer algebra, tensor polynomial simplification including multi-term symmetries, fermions and anti-commuting variables, Clifford algebras and Fierz transformations, component computations, implicit coordinate dependence, multiple index types and many more. The input format is a subset of TeX. Both a commandline and a graphical interface are available.”

This overview shows that Cadabra ranks with other fully-featured programs for CA in GR. The original version is still available but has been superseded by cadabra2. It is written by Kasper Peeters and makes use of sympy and the xPerm module of xAct (see Sect. 6.3.4). There is a substantial user community (as shown by citations) and development work is ongoing.

### Lisp programs for Posets

Rafael Sorkin has written a library of programs to work with posets (partially ordered sets) [C59]. As far as I know the package does not have an overall name. The programs were originally based on elisp, the Lisp underlying the well-known GNU emacs editor program, but are also now available in Common Lisp. The principal application was intended to be work on causal sets, an approach to quantum gravity initiated by Sorkin (Sorkin 1991; Dowker 2013).

### Ortocartan

Ortocartan (earlier called Orthocartan) underwent active development over a considerable period: see for example Krasiński and Perkowski (1981a, b) and Krasiński (1985, 1993, 2001). It was and is principally aimed at calculations in orthonormal frames. Active development was suspended in 2000, at which stage the package ran using the CSL base for Reduce (see Sect. 3.1.4) and would work on (e.g.) Atari Mega STE machines. The code remained obtainable from the principal author, as was the manual. Recently it has been re-implemented by Andrea Magnoni, again using CSL, and the resulting code is now in the “contrib” section of the Reduce source code [C6] and will therefore be automatically downloaded by those installing Reduce.

### Sheep

Sheep’s name is a joke, since it grew out of Ray d’Inverno’s LAM (“lamb”), mentioned in Sect. 1.3. Sheep is a relatively small and fast system (see e.g. De Rop et al. 1984).

Sheep’s first version was written by Frick (1977b) with contributions by Ian Cohen and others, using DEC-10 machine code. It is now written in a dialect of Lisp, Slisp (Sheep Lisp), which is an extension of Standard Lisp. It therefore can be and has been built on the Lisps used by Reduce (see Sect. 3.1.4). Past versions have also used other Lisps: the current version is 062. Because of the shared basis, it is possible to build combined Reduce and Sheep packages (Skea 1989) which enable Sheep calculations to access the general algebraic capabilities of Reduce. By itself, Sheep lacks, for example, polynomial division and integration.

There are two major extensions of the basic Sheep package: Classi, whose principal author is Jan Åman, and Stensor (formerly STENSR) with principal author Lars Hörnfeldt. Classi is a component calculator, with extensive facilities implementing the classification of exact solutions as applied in the “equivalence problem” (see Sect. 8.3.3 and Stephani et al. 2003, Chapt. 9), and Stensor is an indicial calculator. Stensor provides “tensor compiling” so that components of the tensors defined in Stensor can be computed in Sheep or Classi.

Sheep itself initially provided just coordinate component calculations. It has been extended to tetrads of all kinds, and Newman–Penrose spinor calculations, in a very flexible manner. Most of the extra component calculation facilities are part of Classi rather than bare Sheep (Frick and Åman 1985). The first article in MacCallum et al. (1994) provides a still valid introduction to Sheep, Classi and Stensor, although the code has been amended and extended since.

Within Sheep, Classi and Stensor have extensive facilities for defining new tensors in both component and indicial forms, making use of their symmetries, handling co- and contra-variant contractions, switching between bases and coordinate systems and so on. Classi has many spinor facilities. Stensor can represent (e.g.) splits of spacetime and internal manifolds of a fibre bundle. Sheep is interactive and has good facilities for making targeted substitutions. For instance one can specify a substitution to be used only when “printing” (to the screen) so that one can try different substitutions without affecting the stored values.

The main hurdle for new users learning Sheep is that the interface language, although it is reasonably easy to learn, is in the usual Lisp format, rather than the type of interface language used for the other CA systems and packages described above. Formulae are input in a typical Fortran-like style, and the output can be viewed either in the same manner, or like the example in Sect. 4, or using a TeX-like interface.

Sheep has been used not only in general relativity, but also in elasticity theory (Mouton 1978), gauge theories and supergravity, thermodynamics, and fluid dynamics. Examples of the GR applications are given in Sect. 8.

### Earlier systems

#### POLYNOM

POLYNOM (Hoenselaers 1982a, b) was written in order to handle the very large polynomials that arise in stationary axisymmetric exact solutions. It was described at length and used in Hoenselaers’ habilitation thesis (private communication). Its implementation language was ALGOL-W and it made use of a non-standard way of dealing with polynomial representation.

#### Tensign

This program, written in C by Anders Höglund, enabled one to (for example) use the rule relating the Weyl and Riemann tensors to pass from one to the other. Its interface enabled use of a cursor to choose where an operation should be performed. It is still available from the author (at andersh@lysator.liu.se), although he now works in another field.

One could add SCHOONSCHIP (Veltman and Williams 1993) to this list because it has been used in quantum GR (some examples are mentioned below). It is considered superseded by FORM.

## Applications

In this section, areas of application known to me are summarized, but the examples and references given here are just a few of many (each of the bibliographies cited above for the more widely-used packages contains hundreds of papers, among them large numbers of papers in GR). The applications mentioned are thus necessarily a personal selection. (Note to readers: if you are expert in those areas of GR furthest from my own research and expertise, I would welcome suggestions for an improved selection.) Trying to cover a wide field necessitates rather brief descriptions of papers. Cross-references to packages used are shown as described in Sect. 1.5. I have tried to select both early examples and more recent ones.

In theoretical physics the highest level of endeavour, but the least frequently achieved, is to invent a widely-accepted new theory.

Within an established theory one can try to prove a general result (like the famous singularity theorems), or obtain results by approximation techniques (including numerical methods), which is one of the main activities of applied mathematicians, or seek exact solutions. Justifying approximations in nonlinear theories can be difficult: one can seek to prove correctness either by a priori justification through, for example, error estimates, or by comparison of the results with expectations on other grounds.

Finding exact solutions is also non-trivial in highly nonlinear theories like those of relativistic gravity, and can only be done in special cases, but can be useful in several ways: the solutions may be good approximations to physically important situations, they may assist in studies of the general properties of the theory, and they can be used as tests of numerical schemes.

All those activities can be supported by CA methods. One can use CA in obtaining general theorems e.g. for differential identities (MacCallum et al. 1994 {Sheep}). CA can be used for approximation techniques, e.g. in power series expansions, stability analyses, and asymptotics (Piper 1997b; Chruściel et al. 1995 {Sheep}), and CA has long been used (Nakamura 1987) in numerical relativity to generate formulae for numerical evaluation.

Modern methods in CA can be brought to bear on GR problems. Gröbner basis methods were used by Carminati and McLenaghan (1987) for investigating Huygens’ principle, Caprasse et al. (1991) {Reduce’s GROEBNER module} for fourth-order gravity in higher dimensions, and Hartley and Tuckey (1995) for studying Clifford algebras and Grassmannians. Wu’s method was used in Åman et al. (1991). Marcelo Araujo and I (unpublished) looked for closed form quasi-normal modes of black holes using a variant of Kovacic’s (1986) method for finding Liouvillian solutions of second order linear differential equations.

Much of CA in GR work is focused on general relativity, but more recently higher-dimensional and modified classical theories of gravity have also been treated. CA systems, including ones not designed for curved manifolds, have also been used in quantum GR.

### General results in GR

Most of the applications that belong under this heading consist of the generation and analysis of systems of equations for particular classes of problem.

Bruni et al. (1995) {Maple} studied the general dynamics of irrotational “silent universes”, cosmological models in which the magnetic part of the Weyl tensor, $H_{ab}$, vanishes and there are no non-local influences on local dynamics (Dray 1996).

Black hole thermodynamics has an internal geometry that can be studied using differential geometric programs. There are a series of papers on this (e.g. Åman et al. 2003, 2015 {Sheep}), which have also considered higher-dimensional cases (Åman and Pidokrajt 2006 {Sheep}).

As noted in Sect. 4, one can use component calculus to generate generic expressions with symbolic values for (e.g.) the NP spin coefficients as a step in an investigation. Such calculations have been made by a number of people, for example for the orthonormal tetrad ($3+1$ spacelike and timelike) and NP equations (by Åman and myself in Sheep, with results used in Stephani et al. 2003), for NP by Campbell and Wainwright (1977) {CAMAL, checked with Macsyma} and by McLenaghan et al. (see McLenaghan’s article in MacCallum et al. 1994 {Maple}), and for equations in a general tetrad in Holmes et al. (1990) {Sheep}.

Among other formalisms for which CA programs have been developed are the ADM (Arnowitt–Deser–Misner) formalism for the Cauchy problem based on a $3+1$ decomposition into space and time variables and the resulting Hamiltonian formalism (see De Rop et al. 1984; Moussiaux et al. 1983; Tombal and Moussiaux 1985 {Reduce, Sheep, Macsyma}), and the Ashtekar formalism rewriting the Einstein equations in terms of a spin connection (Giannopoulos and Daftardar 1992 {Stensor}).

Calculating the Lanczos potential for the Weyl tensor is quite complicated and so is another natural area for CA’s use: see Dolan and Muratori (1997, 1998) and Edgar and Höglund (1997) {Sheep, Maple}.

d’Inverno (1980) reported how use of functional differentiation for the Noether identities in the Bondi metric led eventually to the development of the $2+2$ formalism for the field equations, of use in theoretical and numerical work on gravitational radiation and for work in spacetimes where two-surfaces naturally arise, e.g. Brizuela et al. (2006).

Torre (2014) {DifferentialGeometry} found conditions for a geometry to admit a null electromagnetic field, thus resolving a problem that had not been completely solved at the time of Stephani et al. (2003). In Krongos and Torre (2015) the results were extended to perfect fluids and scalar fields, and to include a cosmological constant.

Galaev (2014) {DifferentialGeometry} showed how one can find decompositions of Riemannian and Lorentzian manifolds which enable computation of their connected holonomy groups.

#### Uses of indicial calculations

Indicial tensor calculus is used in many theoretical investigations.

One problem which involves both the issues for indicial tensor calculus mentioned in Sect. 4 is that of enumerating scalar polynomial invariants (SPIs) in the Riemann tensor and its derivatives. A number of CA programs to calculate SPIs have been developed over the years, starting quite early (e.g. Hörnfeldt and Pavelle 1983 {Macsyma}). While the maximum number of independent SPIs at each order of differentiation of the Riemann tensor in general relativity has long been known, one cannot give a list of exactly that number which suffices to give all independent SPIs in all cases (for a review see MacCallum 2015). For the undifferentiated Riemann tensor, the slightly larger set defined by Carminati and McLenaghan (1991), which will always include all independent SPIs, can be used.

The work of Fulling et al. (1992), aimed at possible Lagrangians for gravity theories, provides one reason for interest in the problem. In their paper, Fulling et al. “(i) determine the dimensions of the spaces generated by each set of homogeneous monomials formed by multiplication and contraction of the Riemann tensor and its derivatives up to order twelve for scalars; (ii) construct bases of the above spaces up to order eight for scalars and order six for tensors; and (iii) discuss the design of an algorithm for expressing an arbitrary element of the space in terms of its basis.”

Many of the indicial calculation packages mentioned in Sect. 4, with varying approaches, were applied to this problem: for example the rule-based methods of MathTensor (Christensen 1998), and the brute force approach of Invar (Martín-García et al. 2007, 2008 {Mathematica and Maple}).

In Invar, a list of scalar polynomial invariants (SPIs) of the Riemann tensor was first generated and then the relations between them following from (1), the second Bianchi identity, and dimensional and signature dependent identities were found by exhaustion. Dimensional identities arise from the syzygies explored in the four-dimensional case by Harvey (1995) {Stensor} (see also Hörnfeldt 1990): a more extended study of syzygies was made by Carminati and Lim (see Lim and Carminati 2007), who used graph-theoretic methods. In Invar, the resulting relations are tabulated in a database, available as the supplementary material to program ADZK at [C71], which Invar can search to express any given monomial in terms of a base of all the monomials.

An algorithmic method of expressing SPIs in terms of a basis, and more generally of writing multiterm expressions in terms of a basis, is described in Green et al. (2005), Appendix A, using Young tableaux methods.

Balfagón and Jaén (2000) used the indicial capabilities of TTC to check and correct earlier work finding all possible superenergy tensors, i.e. four-index divergence-free tensors quadratic in the Riemann tensor: a uniqueness property of the Bel–Robinson tensor is proved. Subsequently García-Parrado Gómez-Lobo (2008) {xAct} used indicial computing in studying the Bel-Robinson tensor’s dynamics, treated by making a 3+1 split into parts analogous to those which arise for an electromagnetic field obeying Maxwell’s equations, and later (García-Parrado Gómez-Lobo 2014 {xAct}) found a new formulation of a conservation law for it.

In a series of papers, (Lie) symmetries and related concepts for wave equations of various spins have been studied, in particular on vacuum Petrov type D backgrounds, using xAct. Andersson et al. (2014) treated the conformal wave, Dirac–Weyl and Maxwell equations on a general spacetime using the NP formalism, while Aksteiner and Bäckdahl (2016) studied (Lie) symmetries of the system of differential equations for spin 1 and spin 2 fields on a vacuum type D background, confirming earlier results and finding new operators, using the GHP formalism. (There are quite a number of packages for finding such symmetries which have been applied in GR: some are mentioned elsewhere in this review.) The identities and operators found are relevant to separability of the equations, conservation laws and decay rates of test or perturbative fields (Aksteiner et al. 2017).

Cadabra is cited in ten or more papers per year, many in areas related to quantum gravity: examples are given by Buchel et al. (2008) and Butter et al. (2017). A full list can be obtained via [C56].

#### Alternative gravity theories

An early use of CA in GR was to analyse alternative classical gravity theories, e.g. Pavelle (1978) {Macsyma}. Pavelle (1979) describes finding conserved quantities for higher-order gravity Lagrangians (see also Sect. 6.4.3).

A number of authors have used CA in studying theories with torsion and/or nonmetricity. Petti (2016) {Macsyma} discussed derivations of Einstein–Cartan theory from general relativity, extending ideas first put forward in Petti (1986). The extension of component calculations from (pseudo-)Riemannian spacetimes to spacetimes with torsion, such as those in Einstein–Cartan theory, was carried out in Sheep/Classi and used in studies of classification and equivalence of solutions (Rebouças and Åman 1987; Fonseca Neto et al. 1992, 1996). Reduce and EXCALC were used in a series of investigations of Poincaré gauge theory: see McCrea’s contribution in MacCallum et al. (1994) and Baekler et al. (1988), for example.

Magnano et al. (1990) {Stensor} studied the general dynamics of higher-derivative gravity theories and Capozziello and Stabile (2009) {PROCRUSTES} the Newtonian limit of fourth-order theories arising from quadratic Lagrangians.

A number of modern systems (for example, Sheep) allow the user to set the dimension, which could be more or less than 4, and may also go beyond Riemannian spacetimes of higher dimension by enabling calculations on the auxiliary fields arising in alternative theories of gravity (alternative, that is, to general relativity) or unified theories. Some of the systems enable Kaluza–Klein splitting or can handle quantum gravity in higher dimensions.

For examples of work in 3 (i.e. $2+1$) dimensions see Barrow et al. (1986) {Sheep} and Krongos and Torre (2017) {Differential Geometry}. Examples of use in higher-dimensional theories (i.e. higher than 4) are given by De Rop and Demaret (1988), Demaret et al. (1990) and Socorro et al. (1998) {Excalc}; Caprasse et al. (1991) cited above; Shaker-Jomaa (1985) {Sheep} and Birkandan (2008).

Anderson et al. (2015) {DifferentialGeometry} studied the holonomy of ambient metrics: for a given metric $g_0(x^i)$ in dimension *d*, these are Ricci-flat metrics of dimension $(d+2)$ of the form

35 $$\begin{aligned} \tilde{g} = {\mathrm{d}}t\, {\mathrm{d}}(\rho t) + t^2g\left( x^i, \rho \right) , \end{aligned}$$

where $g(x^i, \rho ) \rightarrow g_0(x^i)$ as $\rho \rightarrow 0$, which are Ricci-flat. A large class with holonomy equal to the exceptional non-compact Lie group $G_2$ were found, and the examples include conformal pp-waves.

Farina Busto (1988) {Reduce, Sheep} studied quadratic Lagrangians in a $D+d+1$-dimensional space. A C++ program not listed above was created for five-dimensional braneworld theory by Martin et al. (2005).

Sorkin’s work on causal set theory has already been mentioned, see Sect. 7.2.

A Finsler space package is listed above (see Sect. 6.2): for an application see Antonelli et al. (2003). Other applications of CA to Finsler spaces have been reported by d’Inverno (1980), Rutz (1998) {Reduce} and García-Parrado Gómez-Lobo and Minguzzi (2016) {xAct}.

#### Quantum GR

Hartley et al. (1991) {Reduce} gave a rather general discussion of using CA for constrained dynamical systems following Dirac’s approach, of relevance to canonical approaches to quantum gravity.

Christensen (1998) {MathTensor} reported work to support DeWitt’s approach to quantum field theory in curved spacetime, which required calculation to high orders of the behaviour of the geodesic interval $\sigma$ as $\sigma \rightarrow 0$. This was also addressed by Rodionov and Taranov (1987, 1988a) {Reduce} and by Fulling (1991) who used bespoke C programs.

Capper and Dulwich (1983) used SCHOONSCHIP to study off-shell quantum gravity, while van de Ven (1992) {FORM} computed two-loop terms. Álvarez et al. (2015) {xAct} also considered loop corrections, in another context. One of the authors (Herrero-Valea, private communication) commented that “In general, computing loop corrections in quantum gravity requires to handle tensorial extensions with up to eight indices and internal symmetries”.

Gies et al. (2015) {xTras} analysed “a two-parameter class of covariant gauge conditions, the role of momentum-dependent field rescalings and a class of field parametrizations.” This involved the York decomposition of the 3+1 formalism and “extensive tensor calculus”.

Einhorn and Jones (2015) {xTras} showed that for quantum field theories involving gravity that are classically scale-invariant, “gravitational radiative corrections are crucial in the determination of the nature of the vacuum state in such theories, which are renormalizable, technically natural, and can be asymptotically free in all dimensionless couplings.” xTras was also used in studies of higher-spin fields in an (A)dS background: Boulanger et al. (2013) studied the uniqueness of such theories and Joung and Taronna (2014) cubic interactions in them.

A number of investigations in supergravity, superstrings and supersymmetric field theories have been made using CA. See for example dos Santos (1989), Grimm and Kuhnelt (1980), Amerighi et al. (1986), Hughes and King (1987), dos Santos and Srivastava (1989), Gray et al. (2012) {Stensor, Reduce, EDC} and Cecchini and Tarlini (1990) using Lisp, and papers listed at [C56]. There are also specialized packages for this application not listed earlier: e.g. Kreuzberger et al. (1990), Lucic (1995), Krivonos and Thielemans (1996), Gray et al. (2009) and Gurin (1989) used SCHOONSCHIP.

An application to four-dimensional conformal gravity theories was reported by Irakleidou et al. (2015) {xAct and RTGC}.

Dunajski et al. (2013) used DifferentialGeometry in lifting solutions of Euclidean Einstein–Maxwell equations with non zero cosmological constant to solutions of eleven-dimensional supergravity theory with nonzero fluxes.

Demichev and Rodionov (1985) {TAVI} studied higher-dimensional theories of Kaluza–Klein type, giving as an example the standard model of the electroweak interaction and noting the potential application to extended supergravity. See also Demichev and Rodionov (1986). Gibbons et al. (2011) {EDC} found new metrics on compact simple group manifolds.

Gusynin and Kornyak (1999) studied heat kernels and DeWitt–Seeley–Gilkey coefficients, using a program in C. Foakes and Mohammedi (1988) {Stensor} calculated three-loop terms in a nonlinear sigma model, a calculation where, with advanced substitutions and simplification of expressions with many contracted Riemann products, a result with a few terms could be found (Lars Hörnfeldt, private communication).

### Approximation and numerical schemes

There are quite a number of approximation schemes of importance in GR: the post-Newtonian scheme for celestial mechanics and two-body problems including black hole mergers; expansions at asymptotic infinity and their use for gravitational radiation; weak-field approximations; Taylor series for the metric; the velocity-dominated approximation near the big bang; and perturbations of the metric in cosmology with implications for large scale structure.

#### Expansion approximations

The Sheep power series module TPS, written by Matthew Piper, provided the basis for an implementation of the double series approximation (Piper 1997a, b), an expansion in two parameters describing characteristic mass and length scales originally introduced by Bonnor. This was used in investigations of interactions in gravitational wave-tails (Bonnor and Piper 1998) and of the recoil effects of radiation on the “gravitational wave rocket” (Bonnor and Piper 1997).

PROCRUSTES in Maple and EFTtoPNG in Mathematica$^{\textregistered }$ enable post-Newtonian approximations to be calculated (not only in general relativity: see Capozziello and Stabile 2009). Klioner (1998) {EinS} showed how CA could be used to determine a local reference system for the Earth in the Parametrized Post-Newtonian framework, which is key to the analysis of solar system tests of gravity theories.

A number of papers have used xAct to study post-Newtonian effects (see the ‘Articles’ tab of [C38]). Recent examples are given by Bernard et al. (2017) where the dynamics of compact binary systems were studied up to 4th post-Newtonian order in harmonic coordinates and Marchand et al. (2016) where non-linear backscatter in gravitational wave tails was calculated to 4.5th post-Newtonian order and compared with black hole perturbation calculations.

Poisson and Douçot (2017) studied tidal currents in rotating neutron stars in a post-Newtonian setting. Poisson has said that the use of GRtensorIII was essential in the work.

In general relativity and other GR research, the asymptotic behaviour of spacetime at large distances may be of physical importance (e.g. in studying energy loss by radiation). Expansion in multipole moments for stationary axisymmetric metrics was studied in several papers (Koppel and Ikhermann 1988; Fodor et al. 1989 {Reduce}). ALAM was used for the power series expansion of the Bondi metric (d’Inverno 1983) to 8th order in the luminosity variable. The expansion of this metric in the more general “polyhomogeneous” case was carried out in Chruściel et al. (1995) {Sheep}.

Following that work, Chruściel et al. (1998) {Sheep} proved the uniqueness of the Trautman–Bondi energy, even in the polyhomogenous case, among functionals within a natural class that are monotonic in time for all solutions of the vacuum Einstein equations admitting a smooth “piece” of conformal null infinity  that have passive BMS invariance.

In a series of papers Valiente Kroon used Maple to study expansions near null and spatial infinity, finding obstructions to the smoothness of null infinity and prompting the conjecture that this requires Schwarzschildian initial data near infinity in an appropriate sense (see e.g. Valiente Kroon 2005); most recently, this work has yielded candidate solutions which lack the well-known peeling property (Gasperin and Valiente Kroon 2017 {xAct}). Another set of papers by Valiente Kroon and collaborators has focused on the characterization of initial data giving rise to (e.g.) the Schwarzschild and Kerr solutions: see e.g. Bäckdahl and Valiente Kroon (2010) and García-Parrado Gómez-Lobo (2016) {xAct}.

The generalization of the asymptotic techniques to spacetimes with a positive cosmological constant has been studied and applied to compute radiation from a binary system in such a background (Bonga and Hazboun 2017 {DifferentialGeometry}).

An early example of CA for an approximation scheme was Synge’s method (Åman 1977, 1982 {Sheep}). The approximation is a weak field expansion in the magnitude of the energy-momentum. The CA calculation discovered an error in the hand calculation of McCrea (1973).

CA has also been used to produce Taylor series expansions for the metric, an idea proposed by Penrose and described in Penrose and Rindler (1984). The expansion in Riemann normal coordinates has been done with a special FORTRAN program (Yamashita 1984). A spinorial version of such series, applied to expansion of the Weyl tensor about the apex of a light cone, was introduced by Frauendiener and Sparling (1993): they call it the “non-commutative Newman–Penrose formalism”, but do not mention an implementation.

Holmes et al. (1990) {Sheep} is the only example of CA applied specifically to the velocity-dominated approximation that I know of. Jakubi (1998) {Maple} computed power-series expansions of homogeneous cosmological models for use at very early or very late times.

Cusin et al. (2017) {xAct} computed the power spectrum of the vorticity and rotational velocity to second order in perturbations of a dark matter fluid with non-zero velocity dispersion.

#### Perturbations and stability

Perturbation theory is a natural area of application (and its methods overlap with the expansions just discussed). CA has been applied to studying pulsations and their stability from a very early stage. Frick (1977a) reported a perturbation calculation using Levy’s metric for slowly pulsating axisymmetric systems (Levy 1968), implemented by truncated power series in Sheep. CA was used to study the stability of Melvin’s universe (Safko 1968) and in studies of slowly rotating stars by Thorne and Campolattaro (1967) and Hartle (1967) (as a check on hand calculations). It has recently been used in new studies of the same problem (Reina and Vera 2015 {Reduce}). The perturbed matching technique used there has also been applied to the I-Love-Q relations for neutron star binaries, resolving some discrepancies in earlier calculations (Reina et al. 2017 {Reduce}).

The xPert and Harmonics modules of xAct, still then in development, were used in Brizuela et al. (2006) to study a problem similar to that treated in Reina and Vera (2015), that of perturbations to second order of a spherical spacetime.

Another recent example of CA for perturbation theory is given by Fodor et al. (2010) {Maple, Mathematica}: here spherically symmetric almost periodic long-living localized objects, called oscillatons, formed by a self-gravitating massive real scalar field, were studied. Expansion in terms of the amplitude parameter was made as high as 6-th order.

Rostworowski used Mathematica to study nonlinear gravitational waves arising in a Regge–Wheeler perturbation expansion of the Einstein equations around some symmetric exact solution (Rostworowski 2017b), and applied the concepts in particular to asymptotically Anti-de Sitter vacua (Rostworowski 2017a), carrying the expansion to third order and obtaining evidence for the existence of time-periodic solutions.

A somewhat similar use of an expansion scheme appears in Martinon et al. (2017) {Maple, Mathematica}, where periodic localized configurations formed by gravitational waves were studied. “Going to higher order in the expansion is necessary in order to decide which of the large number of linear solutions correspond to valid nonlinear solutions of the system.”

Lake (1998) mentions some work of Davies on black hole perturbations, allowing the “second order Zerilli function” to be computed, but I have not found a write-up of this.

Unruh (1998) {GRtensorII} applied CA to long wavelength cosmological perturbations, clarifying some earlier work and the then current disagreements. Malik and Wands (2009), in a detailed review of cosmological perturbations, used GRtensorII and Cadabra. Pitrou et al. (2013) have developed and applied a Mathematica$^{\textregistered }$ module within xAct, xPand, to carry out perturbations of homogeneous cosmological models. A number of papers have made use of this module, for example to study the HI brightness temperature up to third order (Umeh 2017).

Lagos et al. (2016) developed a new module xIST, on xAct, which provides a framework for linearized perturbations of spatially homogeneous and isotropic cosmologies in scalar-tensor theories, and applied it. This was an example of a general method for such perturbations, which was also applied to vector-tensor theories.

Sedin (2016) {RTGC} studied the stability of discrete cosmologies.

#### Interfacing to numerical work

CA programs can interact with numerical programs in three main ways; they can be used to replace a numerical program or part thereof, to analyse a numerical program, or to generate a numerical program.

The first of these is valuable not only when the algebra program can give a complete rather than approximate solution, but also when it can give an exact answer to a part of a problem which is numerically ill-conditioned or where it can avoid repetition of a slower numerical calculation (e.g. Autin and Bengtsson 1989). I do not know any direct examples in GR, although analytic solutions have long been used to test numerical schemes (see e.g. Centrella et al. 1986), which is in a way the same issue.

Algebraic analysis of numerical schemes can tackle various aspects. It can enable one to discover instabilities (e.g. the problems shown by Eastwood and Arter 1986 were found using Macsyma) or perform error estimates (see Mrozek 1996 and the special issue of *J. Symb. Comput.*  vol. 24) or grid generation. Exact solutions of problems to be tackled numerically can be important in testing codes.

Gundlach and Martín-García (2004) {xAct} considered two variants of the ADM formalism that admitted a complete set of characteristic variables and a conserved energy that can be expressed in terms of the characteristic variables, the BSSN and NOR formalisms. They showed the systems were symmetric hyperbolic and proposed a family of constraint-preserving boundary conditions that is applicable if the boundary is smooth with tangential shift. In a subsequent paper (Gundlach and Martín-García 2006 {xAct}) they found when the formalisms are equivalent and proposed a modification to BSSN to ensure it was always strongly hyperbolic.

Okounkova et al. (2017) {xAct} used CA to perform calculations leading to their numerical simulations of binary black hole mergers in gravity theories which have general relativity as a limit (applied to Chern-Simons theory in this case).

Generation of numerical code by CA systems has focussed on the production, via templates and algebraic generation of formulae, of FORTRAN or C programs. At the simplest level one can generate assignment statements for the target language. A second level is program templates. The third is optimization of the resulting code (as in the SCOPE package of Reduce) by use of intermediate expressions and common sub-expression searches. Finally, good programs can come close to real code generation including automatic type declarations, selection of numerical algorithms (Dewar 1992) and the other features mentioned above in combination (Cook 1992; Borst et al. 1994). Kranc (see above) is the principal current example known to me of such programs in GR.

### Exact solutions

CA systems and packages, particularly the ones of component calculus type, have perhaps most frequently been used in gravity theory for exact solutions, not only for checking them, but also finding them, and giving unique characterizations.

#### Checking solutions and computing their properties

The simplest and perhaps most common use of CA for exact solutions is to check correctness, not only for vacuum solutions but for non-vacua where the matter field equations and their properties come into play. To do so one might need facilities to implement coordinate or tetrad changes or for changing the sign conventions. This use of CA in GR is very common but frequently not acknowledged. Some early examples appear in d’Inverno and Russell-Clark (1971) and Gibbons and Russell-Clark (1973), and d’Inverno (1980) lists many more. Recent texts on exact solutions have used CA in their preparatory work, e.g. Krasiński (1997) {Ortocartan} and Stephani et al. (2003) {Classi}.

One interesting development in this area is the advent of online databases of solutions. Databases of the solutions in the first edition of Stephani et al. (2003), aimed at providing the classifying information needed to resolve the equivalence problem as described in Sect. 8.3.3 below, were prepared by Skea and colleagues using methods provided in Sheep and Maple (MacCallum et al. 1994; Pollney et al. 2000a; d’Inverno 1998) [C73], and in a different form by Lake and colleagues (Ishak and Lake 2002) [C74] using GRTensorII (see Sect. 6.2.2). A majority of the solutions in Stephani et al. (2003) had been checked using Classi, but the files were not brought to a form fit to make public. More recently Ian Anderson and Charles Torre (private communication) have checked all those solutions using DifferentialGeometry (see Sect. 6.2): a first version of their database is included in the Maple physics package and the full set will be posted at [C20].

Although some packages described above offer little more than the basic calculation of the curvature from the metric in a coordinate basis, the “metric application” (d’Inverno 1983), many also offer calculations in frames (mostly orthonormal or NP) or spinors.

Having obtained the curvature, one may want to study its structure, for example calculating the Petrov type of the Weyl tensor, first done using LAM (d’Inverno and Russell-Clark 1971), or the Plebanski–Petrov or Segre type of the Ricci tensor (Joly and MacCallum 1990; Seixas 1991). Variants of the original algorithms, aimed at improved efficiency, have been discussed in several subsequent papers (see Hon 1975; Letniowski and McLenaghan 1988; Åman et al. 1991; Piper 1997b; Pollney et al. 2000c; Zakhary et al. 2003; Zakhary and Carminati 2004 {Reduce,Maple,Classi} and references therein).

Methods for finding and working with Killing, homothetic or other symmetries have been used in a number of papers. CA can check (Joly 1987 {Sheep}) or find a metric’s isometry group (Karlhede and MacCallum 1982; Araujo and Skea 1988a, b; Araujo et al. 1992; Grebot and Wolf 1994 {Sheep, Reduce}) and other symmetries such as homothety (McIntosh and Steele 1991; Koutras and Skea 1998; Vaz and Collinson 1993 {Sheep, Reduce}) or conformal motions. Some applications are described in McLenaghan and van den Bergh (1993) {Maple}, O’Connor and Prince (1998) and Hickman and Yazdan (2017) {Dimsym, Exterior} and in the next subsection. See also Sect. 8.3.3.

The CRACK package (distributed with Reduce), which was written to handle over-determined systems of partial differential equations, can be used to investigate the existence of symmetries and various other problems giving similar systems of equations (Grebot and Wolf 1994; Wolf 1996, 1998). There are quite a number of other packages addressing similar problems, and able to find symmetries of differential equations and systems, but no recent comprehensive review of them that I know of. Others used in examples in this review are Dimsym in Reduce and Exterior in Maple.

The physical interpretation of solutions is very important and can also be aided by CA, see e.g. Delgaty and Lake (1998) {GRtensorII}.

Properties of fields in spacetime have also been studied using CA. McLenaghan and collaborators used Maple’s NP packages and Gröbner bases to characterize those spacetimes in which scalar, neutrino and Maxwell fields obey Huygens’ principle: see for example McLenaghan’s article in MacCallum et al. (1994) and McLenaghan and Sasse (1996).

One can use CA to study curves within the metric, e.g. obtain the geodesic equations (and perhaps try to solve them if the system has a good differential equations solver). Prince and Sherring constructed Reduce and EXCALC programs to investigate the symmetries of geodesics and of the tangent bundles in Lagrangian mechanics (Prince 1988a, b; Prince and Sherring 1988a, b).

Using invariants calculated by CA to locate and characterize singularities and horizons has been discussed quite often. Gibbons and Russell-Clark (1973) found a naked singularity, but no horizon, in the Tomimatsu-Sato solution. Karlhede et al. (1982) {Sheep} showed that a certain Riemann invariant located the Schwarzschild horizon; Skea (1986) then showed the same invariant did not characterize other horizons. MacCallum (2006) {Classi} offered an improved criterion and considered further cases. More recently, both scalar polynomial and Cartan invariants, as defined in e.g. MacCallum (2015), have been used (Abdelqader and Lake 2015; Page and Shoom 2015; Brooks et al. 2018 {GRTensorII, Sheep, Reduce}): the last of these papers considers some five-dimensional examples.

These techniques also apply to manifolds of other dimensions or signatures. For examples see the work of Birkandan (2008) in Euclidean signature and Heinicke and Hehl (2015) {Reduce}.

GHP was used (Vu and Carminati 2003) in a study of the shear-free conjecture. The conjecture, supported by the large range of more special sets of conditions for which it has been proved, is that a general shearfree perfect fluid will have either zero expansion or zero vorticity. CAMAL (Collins and Wainwright 1983) and Maple’s forms package have also been used in studies of this problem (Lang 1993), most recently addressed in a series of papers by Carminati, Huf, Karamian, Vu and van den Bergh in various combinations (see e.g. Carminati 2015; Huf and Carminati 2018 {TensorPack}).

The embedding problem (see Stephani et al. 2003, Chapt. 37) has also been approached using CA (Roque and dos Santos 1991 {Reduce}).

#### Finding new solutions

Most systems providing the metric application can also be used as a workpad to explore hypotheses on the metric or the curvature: for examples see Czapor (1995) and Bradley and Sviestins (1984) {tensor in Maple, Reduce}. An amusing possibility is described in Hoenselaers and Skea (1989) {Sheep}. (I have found a couple more solutions by similar happy accidents: it appears this is especially easy to do when the particular field equations have linearity properties.)

Two more serious systematic ways of finding new solutions have been used. One is to invert the unique characterizations provided by the equivalence problem (the next section outlines the problem and work on it): see Karlhede and Lindström (1983) and Bradley and Karlhede (1990) {Sheep}. The other is to carry out one or other of the generating techniques, in particular those available for solutions with two commuting Killing vectors (for which see Stephani et al. 2003, Chapt. 34); for examples, see Hoenselaers (1981, 1982b), Chen et al. (1983), Moussiaux and Tombal (1983), Bradley and Curir (1989) and Bradley et al. (1991) {POLYNOM, muLISP, Reduce, Sheep}.

In a series of papers Hennig found new solutions in the Gowdy-symmetric class by applying Sibgatullin’s generation method, e.g. Beyer and Hennig (2014) and Hennig (2016): he commented (private communication) that “the final results could only be obtained with an appropriate combination of Maple and Mathematica, which both have their advantages and disadvantages.” These solutions include some with the “spikes” of interest in studies of cosmological singularities. Another recent example is given in Gregoris et al. (2017) {Maple}, where solutions with intersecting “spikes” were generated.

Finding solutions with given symmetries provides another class of applications. Bona (1988) {SMP} found all those dust metrics with certain symmetries which admitted an invariant conformal vector and McIntosh and Steele (1991) {Excalc} found all vacuum Bianchi I metrics with a homothety. Other examples are given by McLenaghan and van den Bergh (1993) {Sheep} and Seixas (1992b) {Sheep}.

#### Characterizing metrics and the equivalence problem

CA in GR has been used extensively in finding invariant characterizations of metrics. For example, Wainwright (1977) and Szafron and Wainwright (1977) applied the NP programs in CAMAL to study the Szekeres solutions and their generalization. More complete characterizations are used in resolving the equivalence problem.

The equivalence problem is that of identifying (regions of) two geometries that are locally isometric but expressed in different coordinates. Its resolution has been a driver for CA in GR from early on. We now have a well-defined procedure which (for suitably smooth regions) can be used to study the problem by computing a set of quantities uniquely characterizing the manifold (locally) and then comparing characterizations. However, because the comparison step of that procedure would require determination of the compatibility of sets of equations, it remains formally undecidable as a result of the no-go theorems on simplification (Buchberger and Loos 1983). Further arguments for undecidability were given by Kreinovich (1991).

Nevertheless, for practical examples the procedure can be completed. The Classi branch of Sheep was designed for this process (see Åman and Karlhede 1980, 1981) and it was, for example, highly effective in preparing Stephani et al. (2003). Chapter 9 of Stephani et al. (2003) sets out the procedure. For details of implementations see the first article in MacCallum et al. (1994) {Classi} and Pollney et al. (2000a) {GRtensorII}. Apart from these two programs I am not aware of any that systematically compute and record all the necessary quantities. The Classi version is formulated using the NP spinor formalism, and requires the computation of the Cartan invariants of each spacetime: these uniquely determine the spacetime, locally, and comparing values for apparently different solutions gives the way to resolve equivalence. For examples of classification see Pollney et al. (2000c), [C73] and [C74].

Certain families of solutions present special difficulties for this process, notably the infinite set of solutions, with indefinitely many terms in the metrics, which can in principle be obtained by generating techniques. This difficulty can be made less severe (Seixas 1992a {Classi}) by exploiting the factorizations available in many cases (Hoenselaers and Perjés 1990; Hoenselaers 1997) to reduce the sizes of the computations. Other practical difficulties are discussed by Pollney et al. (2000a, b).

More recently it has been argued (Coley et al. 2009) that SPIs also provide sufficient information to characterize spacetimes locally, unless the spacetime is a member of Kundt’s class or its higher-dimensional counterparts, for which the Cartan invariants can continue to be used. The result implies that programs, such as those discussed above, that systematically calculate SPIs can be useful in this context as well. To discuss this conclusion and its implications fully would take us too far afield: see MacCallum (2015) for a review. Many of the papers cited in that review and in its forthcoming extended version use CA to compute invariants.

The methods for the equivalence problem have had a number of consequences. For example they can be used in invariantly defining limits of families of spacetimes (Paiva et al. 1993), in studying junction conditions (Cox 2003 {Classi}) and in the methods for finding the symmetry of solutions mentioned above.

These ideas can be carried over to alternative signatures (Karlhede 1986a), to other theories using connections and curvatures, i.e. general gauge theories (Karlhede 1986b), and to higher-dimensional theories (McNutt et al. 2017; Brooks et al. 2018), although in the last of these cases one may encounter problems in the frame fixing needed for canonical forms of the Cartan invariants, due to the absence of solutions in radicals or other convenient closed forms for quintics and higher degree equations.

## Concluding remarks

To make enough use of each of the systems and packages listed above to enable one to make comprehensive comparisons would be a Herculean task. Moreover it would inevitably be subject to the cautionary notes and possible biases stated in Sect. 1.4. I have not attempted it, confining myself to checking what seemed to me the most important special purpose systems and packages for general purpose systems. My choice of examples, and my comments on systems and packages, are thus unlikely to be unbiased. (I shall be glad to receive, for inclusion in later versions of this Living Review, information on points misrepresented, systems overlooked, and other suggestions that might counteract any resulting imbalances.) Reader, beware!

That said, I shall offer some guidance.

If a new user of CA in GR seeks facilities suitable for his or her problem, a first place to look is in those fully-featured systems which offer both indicial and component calculation. That those are also the most frequently referenced in Sect. 8 may not be just a reflection of a personal bias, although they are the ones I do use, or would use, myself. Currently I would consider the following as being in that category: xAct in

Mathematica$^{\textregistered }$ (see Sects. 6.3 and 6.3.4), the combined Reduce/Sheep versions (see Sects. 3.1.4 and 7.4) and Cadabra (see Sect. 7.1).

If one does not need indicial tensor calculations, the more fully-featured component calculators, notably the physics and DifferentialGeometry packages of Maple (see Sect. 6.2), GRTensorII and III, and ccgrg, should be considered.

However, it may be that none of these is right for other users. In particular a simple and easy-to-learn system with more limited features, one of the many listed above, may be more suitable. Additionally, one should beware of the syndrome that “if your only tool is a hammer, all problems look like nails”. I again emphasize that *there is no best system*; see Sect. 1.4.

A second approach to choice is to look in Sect. 8 to see if an application listed there is close to what one wishes to do. If so, the software used for that application may be the best choice.

As well as the capabilities of systems and packages, choice may be influenced by cost, by available hardware, by operating systems and other software, by the effort involved in learning the system, or by prior familiarity with a suitable underlying CA system. The cost factor may incline a new user towards one of the free systems which already have substantial facilities for CA in GR such as Reduce, Sheep, or the newer Cadabra or Redberry, or to a system for which he or she is covered by an institutional site licence.

There may be some more ambitious researchers who want to write a new CA for GR package. My initial reaction would be: don’t do it. But it could be that those researchers have ideas for structuring a package which really differ from what has been done before, or that they really need facilities not available in current CA for GR or minor extensions thereof. (Given the large number of existing packages, checking that a facility is really unavailable may take some time!). If I were to embark on such an effort myself, I would try to work either by adding to an existing package, preferably a free one, which has a reasonable size user community so that there is some resilience if bugs or other difficulties are encountered, or, if I needed to write a more extensive and independent package, to do so within an existing free general purpose system (so that as wide a community as possible could use my work). Axiom or Sage could be suitable, for example.

## A Index of systems and packages

See Table 2.

**Table 2:** An alphabetical list of the packages and systems listed in Sects. 3, 6 and 7, stating in which (sub)section(s) further information about the package can be found. It is intended to assist readers of Sect. 8 who wish to find out more about a package used in an application

Package	Section	Package	Section
ALAM	1.3	GRworkbench	6.3/6.3.3
atensor (Maxima)	6.1	itensor	6.1
atensor (Reduce)	6.4	Kranc	6.3
atlas2 (Maple)	6.2	LUCY	6.2.2
atlas2 (Mathematica$^{\textregistered }$)	6.3	Macsyma	3.1.1
Axiom	3.2.1	Maple	3.1.2
bianchi	6.2.2	Mapletensor	6.2.2
BOLTZ (see EVOL)	6.3	Mathematica	3.1.3
Cadabra	7.1	MathGR	6.3
CAMAL	1.3	MathTensor	6.3
Canon	6.2	Maxima	3.1.1
CANTENS	6.4.2	muMATH/muLISP	3.3
Cartan (Maple)	6.2.2	MuPAD	3.2.3
cartan (Maxima)	6.1	muTENSOR	6
CARTAN (Mathematica$^{\textregistered }$)	6.3.2	NP/NPspinor/NPtools	6.2.2
ccgrg	6.3	ORTHOFRAME/oframe	6.2.2
Classi	7.4	ORT(H)OCARTAN	7.3
ctensor	6.1	POLYNOM	7.5.1
Debever	6.2.2	Poset library	7.2
Derive	3.3	PROCRUSTES	6.2.2
Differential Forms	6.3.2	Redberry	3.2.4
DifferentialGeometry	6.2	REDTEN	6.4
diffgeo	6.3	Reduce	3.1.4
diffgeom	3.2.5	RGTC	6.3
difforms	6.2.2	Ricci (Lee)	6.3
Dimsym	6.4	Ricci (Aguirregabiria)	6.3
dummy	6.4	RicciR	6.4
EDC/super EDC	6.3	Riegeom	6.2.2
eds	6.4	Riemann (Maple)	6.2
EFTofPNG	6.3	RIEMANN (MuMATH)	3.3
EinS	6.3	RTENSOR	6.4.2
EinsteinTensor	6.3.2	Sage/SageManifolds	3.2.5
excalc	6.4/6.4.3	Sheep	7.4
EVOL	6.3.2	SMP	3.3
Exterior	6.2	Stensor	7.4
Finsler	6.2	susy2	6.4
fjeforms	6.2.2	Symbolic C++	3.2.6
FORM	3.2.2	TAVI	6.4
FORMAC	3.3	Tensign	7.5.2
forms	6.2.2	Tensor (Maple)	6.2.2
GENRE	6.4.2	Tensor (sympy)	3.2.5
GEOCALC	6.1.2	TensorA	3.2.5
geodesicCOMMENTED	6.3	Tensorcalc, see Riemann	6.2
GREAT	6.3.2	TensoriaCalc	6.3
GRG3.2	6.4	Tensorial	6.3.2
GRLIB	6.4.2	TensorPack	6.2
grt	6.3.2	Tensors	6.3.2
GRTensorII	6.2.2	Tetrad	6.3
GRTensorIII	6.2	TTC	6.3.2
GRtensorM	6.3	xAct	6.3/6.3.4
		xPand/xPerm/xPert/xTras	6.3.4

## B Systems providing extended facilities

This appendix provides a cross-reference for packages or systems that embody some of the specific calculation techniques not available in the majority of other packages. In addition those described in Sect. 9 as fully-featured will provide many if not all of these: for example DifferentialGeometry provides an integrated set of facilities covering most of the features listed below. Packages known to be no longer available are omitted here but “earlier” programs that can still be obtained and may be usable are included. The list has been developed from the package descriptions given above or personal knowledge and is therefore highly likely to be incomplete. Appendix A provides a guide to finding the fuller information on availability of specific packages given in Sects. 6 and 7.

One can assume that all the component calculators enable exact calculation of curvature from a metric in a coordinate basis, and provide, or could easily be adapted to provide, the geodesic equations. Many also offer Petrov classification of the Weyl tensor and Segre classification of the Ricci tensor in general relativity. So here I give only pointers to systems which have implemented further methods: the selection of these methods is a personal one.

General tetrad methods are supported by ctensor in Maxima; Tetrad in Mathematica and Classi. Orthonormal tetrad methods are or were provided by ORTHOFRAME/oframe in Maple; Tensorial in Mathematica$^{\textregistered }$; GRLIB in Reduce; Classi and ORTOCARTAN. The Newman–Penrose formalism has been implemented in a variety of systems and packages. See Campbell and Wainwright (1977), Esteban and Ramos (1990) and Birkandan (2008); GRtensorII and GRtensorIII, NP, NPspinor and NPtools in Maple; RTGC and xAct in Mathematica$^{\textregistered }$; GENRE, GRG3.2 and GRLIB in Reduce; and Classi. The GHP (Geroch et al. 1973) formalism, which has a close relation to the NP formalism (see Stephani et al. 2003, Chapt. 7), has been implemented (see Carminati and Vu (2001), Vu and Carminati (2003) {GHP} and Aksteiner and Bäckdahl (2016) {xAct}), but I am not aware of any implementation of Held’s generalization of GHP.

GRtensorII in Maple was the basis for a junction conditions package (Musgrave and Lake 1996, 1997), also supported by GRTensorIII.

Ashtekar variables were implemented by Giannopoulos and Daftardar (1992) {Reduce}.

Clifford algebra manipulation is supplied by SymbolicC++, atensor and GEOCALC in Maxima, and LUCY in Maple. Grassmannians can be treated by atensor in Maxima; see also under diffgeo in Mathematica$^{\textregistered }$ and Hartmann and Davis (1989).

It seems probable that a number of the packages provide facilities available in dimensions other than 4 and other signatures, and allow objects bearing more than one type of index, but this is not always clearly stated. For example, Sheep has an internal variable DEFAULTDIMENSION!*. Two packages, Park’s “Indicial Notebooks” in Mathematica$^{\textregistered }$ and Sheep’s Stensor, specifically mention Kaluza–Klein type splits. TAVI provided Yang–Mills type fields. Birkandan (2008) discusses instanton fields.

Differential form computations are supported by cartan in Maxima; Differential Forms, EDC, MathTensor and RTGC in Mathematica$^{\textregistered }$; and eds, excalc and GRG3.2 in Reduce.

Treatment of symmetries has been discussed in Sect. 8.3.1.

Unusual aspects of geodesic calculations are supported by geodesicCOMMENTED and GRworkbench in Mathematica$^{\textregistered }$. The first allows one to consider whether a set of equations represent the geodesics of a metric, and the second, and some other more numerical packages listed in Sect. 6.3.3, enable numerical integration and visualization of geodesics.

Power series and other expansion methods were provided by: ALAM (d’Inverno 1975, 1980); in Jakubi (1998) and Piper (1997b) {Sheep}; and by EFTtoPNG, PROCRUSTES, xPert and xPand. See also Holmes et al. (1990) for the velocity-dominated approximation and Chruściel et al. (1995) for asymptotically flat spacetimes.

The indicial tensor packages past and present, in the order in which they appear in Sects. 6 and 7, are: itensor for Macsyma/Maxima; Canon (Manssur and Portugal 2004), Riemann (Portugal and Sautú 1997), TensorPack (Huf and Carminati 2015), Mapletensor (Kavian et al. 1996) and Riegeom (Portugal 2000) in Maple; EinS (Klioner 1998), MathGR (Wang 2013), MathTensor (Parker and Christensen 1994), Ricci, TTC (Balfagón and Jaén 1998, 1999), and xAct in Mathematica$^{\textregistered }$; atensor (Ilyin and Kryukov 1994), dummy (Dresse 1993a, b), CANTENS, RicciR (Kadlecsik 1992) and Rtensor (Rodionov and Taranov 1988a, b, 1989) in Reduce; and the standalone systems Cadabra (Peeters 2007a, b) and Stensor in Sheep (MacCallum et al. 1994), which was also adapted for Macsyma. Excalc and REDTEN in Reduce and GRTensorII in Maple, and possibly others, have some indicial capabilities. Some have been described in more detail above, but I do not know in all cases what methods they use.

Some of these packages use one another. For example, CANTENS uses dummy, and the xPerm module in xAct has, as well as the Mathematica$^{\textregistered }$ code, a faster implementation in C which (Martín-García 2008) the author has made available to other packages: it is used by Cadabra (see Sect. 7.1).

Functional differentiation was first implemented in Macsyma’s itensor, then called ITMS (Bogen and Pavelle 1977), allowing derivation of the field equations from alternative Lagrangians, and in LAM (d’Inverno 1975, 1980). It is available in excalc.

Facilities of use in quantum field theory or quantum gravity are provided by MathTensor in Mathematica$^{\textregistered }$; susy2 in Reduce; FORM; and Stensor, and by some special programs for quantum field theory.

## C URLs referenced in the text

**General purpose systems**

1. Macsyma for PCs running Windows XP or earlier:
  http://www.symbolics-dks.com/
2. Maxima: http://maxima.sourceforge.net
3. Maple (from Maplesoft): http://www.maplesoft.com/products/maple/
4. Mathematica$^{\textregistered }$ (from Wolfram Research):
  http://www.wolfram.com/mathematica/
5. Wolfram|Alpha: https://www.wolframalpha.com/
6. Reduce: http://www.reduce-algebra.com/
7. SageMath: http://www.sagemath.org
8. Axiom: http://www.axiom-developer.org/
9. FriCAS: http://fricas.sourceforge.net
10. Open Axiom: http://open-axiom.org
11. Aldor: http://www.aldor.org/
12. FORM: https://www.nikhef.nl/~form
13. MuPAD, within MATLAB: http://www.mathworks.com/products/symbolic/
14. Redberry: http://redberry.cc
  **Note:** The country code for this site is assigned to the Cocos (Keeling) Islands but it is also used by the unrecognised Turkish Republic of Northern Cyprus. Wikipedia says that the top-level domain (TLD) was marketed ‘to become the “second largest TLD registry in the United States second only to Verisign” according to Brian Cartmell, founder and CEO of eNIC’.
15. SymbolicC++: http://issc.uj.ac.za/symbolic/symbolic.html
16. Xcas: http://www-fourier.ujf-grenoble.fr/~parisse/giac.html

**Maple packages**

17 Maple physics package webinar: http://connect.physicsworld.com/applying-the-power-of-computer-algebra-to-theoretical-physics/2003844.article#webinar
18 Worksheet for the above webinar: http://www.mapleprimes.com/posts/203574-Computer-Algebra-In-Theoretical-Physics
19 atlas2 for Maple: http://digi-area.com/Maple/atlas/
20 DifferentialGeometry: http://digitalcommons.usu.edu/dg/
21 Exterior:
  http://www.math.canterbury.ac.nz/~m.hickman/Exterior/Exterior.shtml
22 GRTensorIII: https://github.com/grtensor/grtensor
23 TensorPack: http://www.bach2roq.com/science/maths/GR/TensorPack.html
24 GRTensorII: http://grtensor.phy.queensu.ca/

**Mathematica** $$^{\textregistered }$$ **packages**

25 atlas2 for Mathematica: http://digi-area.com/Mathematica/atlas/
26 ccgrg in Mathematica: http://library.wolfram.com/infocenter/MathSource/8848/
  Written by Andrzej Woszczyna, Wojciech Czaja, Krzysztof Głód, Zdzisław Golda, Radosław Kycia, Andrzej Odrzywołek, Piotr Plaszczyk, Lech Sokołowski, and Sebastian Szybka.
27 diffgeo: http://people.brandeis.edu/~headrick/Mathematica/index.html
28 EDC: http://www.inp.demokritos.gr/~sbonano/EDC/
29 EFTofPNG: https://sites.google.com/view/levim/public-code
30 EinS: http://rcswww.urz.tu-dresden.de/~klioner/eins.html
31 Kranc: http://kranccode.org/
32 MathGR: https://github.com/tririver/MathGR
33 Ricci (Lee): http://www.math.washington.edu/~lee/Ricci/ Written by John M. Lee with Dale Lear, John Roth, Lee Nave and Larry Peterson.
34 Ricci (Aguirregabiria): see Tetrad below.
35 RTGC: http://www.inp.demokritos.gr/~sbonano/RGTC/
36 TensoriaCalc: http://www.stargazing.net/yizen/Tensoria.html
37 Tetrad: http://tp.lc.ehu.es/jma/math.html
38 xAct: http://www.xact.es
39 FeynCalc: http://feyncalc.github.io
  A package by Rolf Mertig, Frederik Orellana and Vladyslav Shtabovenko, primarily for Feynman diagrams.
40 Park’s Indicial Notebooks:
  http://library.wolfram.com/infocenter/MathSource/755/

**Earlier Mathematica packages**

41 Differential Forms: http://library.wolfram.com/infocenter/MathSource/482/
42 GREAT: http://library.wolfram.com/infocenter/MathSource/4781/
43 EinsteinTensor: http://library.wolfram.com/infocenter/Demos/162/
44 grt: http://www.vaudrevange.com/pascal/grt/
45 Tensorial: http://jfgouyet.fr/Tensorial/IndexT.html
46 Tensors: http://personales.unican.es/castie/tensors/

**Reduce packages**

47 Dimsym:
  http://www.latrobe.edu.au/mathematics-and-statistics/research/geometric-and-algebraic-techniques-for-differential-equations/dimsym
48 RicciR: http://www.kfki.hu/~kadlec/sw/riccir/riccir.tar.gz
49 Redten: http://www.utsc.utoronto.ca/~harper/redten.html
50 GRG 3.2: http://www.maths.qmul.ac.uk/~mm/grg3.2/
51 GRLIB: http://www.maths.qmul.ac.uk/~mm/grlib/

**Sage packages**

52 SageManifolds: http://sagemanifolds.obspm.fr/
53 TensorA: http://www.stat.boogaart.de/tensorA/
54 diffgeom: http://docs.sympy.org/latest/modules/diffgeom.html
55 Tensor (Sympy):
  http://docs.sympy.org/latest/modules/tensor/index.html

**Standalone packages for CA in GR**

56 Cadabra: http://cadabra.science
57 ccgrg in Python: https://pypi.python.org/pypi/GraviPy/0.1.0
58 Sheep: http://www.maths.qmul.ac.uk/~mm/shp/
59 Posets in elisp: http://www.perimeterinstitute.ca/personal/rsorkin/lisp.library/

**Bibliographic and other documentation**

60 Macsyma history: https://en.wikipedia.org/wiki/Macsyma
61 Beebe’s bibliographies: http://ftp.math.utah.edu/~beebe/bibliographies.html
62 swMATH: http://www.swMATH.org
63 Maple book list: http://www.maplesoft.com/books/
64 Mathematica book list: http://www.wolfram.com/books/
65 Wolfram Language and System Documentation Center:
  http://reference.wolfram.com/language/
66 xAct and xTensor bibliography: http://www.xact.es/articles.html
67 Reduce history: http://reduce-algebra.com/reduce40.pdf
68 Reduce Documentation: http://www.reduce-algebra.com/documentation.html

**Other information**

69 CA conferences: http://www.sigsam.org/Resources/ConferenceSeries.html
70 Cython: http://cython.org/
71 CPC program library: http://www.cpc.cs.qub.ac.uk
  Due to changes in publishing arrangements for the parent journal, this URL and the location of the programs are due to change at the end of 2018. The site says old programs will be moved to join those published since 2016 which are in Elsevier’s Mendeley Data library at
  https://data.mendeley.com/datasets/journals/00104655
72 EinsteinToolkit: http://einsteintoolkit.org/
73 Exact solutions database (Sheep, Maple):
  http://www.personal.soton.ac.uk/rdi/database/index.html
74 Exact solutions database (GRTensorII): http://grdb.org
  Note: The server for this experienced a disk crash in September 2017 and the rebuild was still in progress at the time of writing this review.
75 GraviPy: https://pypi.python.org/pypi/GraviPy. Lead author W. Czaja.
76 Gyoto: http://gyoto.obspm.fr/index.html
77 MathML specification: https://www.w3.org/TR/2014/REC-MathML3-20140410/
78 OpenMath: http://www.openmath.org
79 School Mathematics Project: http://www.smpmaths.org.uk/,
80 Wikipedia comparisons page:
  https://en.wikipedia.org/wiki/List_of_computer_algebra_systems

## Footnotes

1 I use the word system for programs that cover a wide area of mathematics, including calculus and algebra, and refer to more specialized programs, or specific application programs within the main systems, as packages. When necessary I shall continue this hierarchy to “modules”: for example Sheep (Sect. 7.4) is a special purpose system which provides two main packages, Classi and Stensor; and Classi provides a module ELDYNF for Maxwell’s equations written in tetrads.

2 Wikipedia is not always accurate, as it is open to being edited by underinformed people, but in my experience it is pretty reliable in mathematical areas.

3 The precise numbers depend somewhat on how the result is formatted.

4 The CAMAL package now available in Reduce supplies only the celestial mechanics capabilities of the original CAMAL.

5 This is far from an exhaustive list: apart from implementations in machine code or assembler for specific machines, other high-level languages such as ALGOL-W and Modula-2 have been used. The recent Redberry (see Sect. 3.2.4) is written in Java.

6 At the extreme, van Hulzen and Calmet (1983) remark that it has been the misfortune of many systems and facilities to be used only at one place, i.e. only by their original author. They say the reasons are often “importability, language design or lack of significance when finally known outside its residence”. Moreover, it has been said that “systems for CA in celestial mechanics are like toothbrushes: one used them every day but would not lend them to friends”. Thus in looking for applications of packages it can help to find the publications of the package’s author(s), which is why I have tried below to list package authors’ names.

7 William Stein, the originator of Sage (described in Sect. 3.2.5) has been quoted as saying “Every great CA package is built on the ashes of an academic career”.

8 Within SageMath, which is not to be confused with the business and finance software Sage, though I shall refer to SageMath just as Sage hereafter.

9 Many systems now allow two-dimensional or more complex input formats, looking more as mathematics usually looks, especially via menu driven graphical user interfaces, “notebooks” or “worksheets”.

10 Stephen Wolfram was awarded the 2017 Richard D. Jenks prize of ACM SIGSAM for his work on Wolfram|Alpha and Mathematica.

11 As of February 2018 the websites for Atlas 2 were no longer reachable, but I have retained the information about Atlas 2 in case this is a temporary glitch.

12 The author of EDC and RTGC, Sotirios Bonanos, died on 1 Oct 2013. The packages remain available, but should perhaps be moved to the “Earlier” category.
